# Systemic Immunomodulatory Treatments for Atopic Dermatitis: Update of a Living Systematic Review and Network Meta-analysis

**DOI:** 10.1001/jamadermatol.2022.0455

**Published:** 2022-03-16

**Authors:** Aaron M. Drucker, Deanna E. Morra, David Prieto-Merino, Alexandra G. Ellis, Zenas Z. N. Yiu, Bram Rochwerg, Sonya Di Giorgio, Bernd W. M. Arents, Tim Burton, Phyllis I. Spuls, Jochen Schmitt, Carsten Flohr

**Affiliations:** 1 Division of Dermatology, Department of Medicine, University of Toronto, Toronto, Ontario, Canada; 2 Department of Medicine and Women’s College Research Institute, Women’s College Hospital, Toronto, Ontario, Canada; 3 Faculty of Medicine, University of Ottawa, Ottawa, Ontario, Canada; 4 Unit for Population-Based Dermatology Research, St John’s Institute of Dermatology, King’s College London and Guy’s and St Thomas’ Hospital, London, United Kingdom; 5 Brown University, Providence, Rhode Island; 6 Dermatology Centre, Salford Royal NHS Foundation Trust, The University of Manchester, Manchester Academic Health Science Centre, NIHR Manchester Biomedical Research Centre, Manchester, United Kingodm; 7 Departments of Medicine and Health Research Methods, Evidence and Impact, McMaster University, Hamilton, Ontario, Canada; 8 Libraries & Collections, King’s College London, London, United Kingodm; 9 Dutch Association for People with Atopic Dermatitis (VMCE), Nijkerk, the Netherlands; 10 Patient Representative (independent), Nottingham, United Kingodm; 11 Department of Dermatology, Amsterdam Public Health/Infection and Immunology, Amsterdam, the Netherlands; 12 Center for Evidence-Based Healthcare, Faculty of Medicine Carl Gustav Carus, Technische Universität (TU) Dresden, Dresden, Germany

## Abstract

**Importance:**

Systemic treatments for atopic dermatitis are being evaluated primarily in placebo-controlled trials; network meta-analysis can provide relative efficacy and safety estimates for treatments that have not been compared head to head.

**Objective:**

To compare reported measures of efficacy and assessments of safety in clinical trials of systemic treatments for atopic dermatitis in a living systematic review and network meta-analysis.

**Data Sources:**

The Cochrane Central Register of Controlled Trials, MEDLINE, Embase, Latin American and Caribbean Health Science Information database, Global Resource of EczemA Trials database, and trial registries were searched through June 15, 2021.

**Study Selection:**

Randomized clinical trials examining 8 or more weeks of treatment with systemic immunomodulatory medications for moderate-to-severe atopic dermatitis were included after screening titles, abstracts, and papers in duplicate.

**Data Extraction and Synthesis:**

Data were abstracted in duplicate. Bayesian network meta-analyses and assessed Grading of Recommendations Assessment, Development and Evaluation certainty of evidence were performed. The updated analysis was completed from June to December 2021.

**Main Outcomes and Measures:**

Outcomes include change in Eczema Area and Severity Index (EASI), Patient Oriented Eczema Measure (POEM), Dermatology Life Quality Index (DLQI), and Peak Pruritus Numeric Rating Scales (PP-NRS).

**Results:**

Since October 2019, 21 new studies were added, for a total of 60 trials with 16 579 patients. Up to 16 weeks of treatment in adults, abrocitinib, 200 mg daily (mean difference [MD], 2.2; 95% credible interval [CrI], 0.2-4.0; high certainty) and upadacitinib, 30 mg daily (MD, 2.7; 95% CrI, 0.6-4.7; high certainty) were associated with reduced EASI slightly more than dupilumab, 600 mg then 300 mg every 2 weeks. Abrocitinib, 100 mg daily (MD, −2.1; 95% CrI, −4.1 to −0.3; high certainty), baricitinib, 4 mg daily (MD, −3.2; 95% CrI, −5.7 to −0.8; high certainty), baricitinib, 2 mg daily (MD, −5.2; 95% CrI, −7.5 to −2.9; high certainty) and tralokinumab, 600 mg then 300 mg every 2 weeks (MD, −3.5; 95% CrI, −5.8 to −1.3; high certainty) were associated with reduced EASI slightly less than dupilumab. There was little or no difference between upadacitinib, 15 mg daily, and dupilumab (MD, 0.2; 95% CrI, −1.9 to 2.2; high certainty). The pattern of results was similar for POEM, DLQI, and PP-NRS.

**Conclusions and Relevance:**

In this systematic review and meta-analysis, abrocitinib, 200 mg; and upadacitinib, 30 mg daily, were associated with slightly better scores than dupilumab, and upadacitinib, 15 mg daily, was associated with similar scores to dupilumab. Abrocitinib, 100 mg daily, baricitinib, 4 mg and 2 mg daily, and tralokinumab, 300 mg, every 2 weeks were associated with slightly worse scores.

## Key Points

**Question:**

What is the association of systemic treatments for atopic dermatitis with Eczema Area and Severity Index (EASI) scores?

**Findings:**

This systematic review with meta-analysis found that compared with dupilumab, abrocitinib, 200 mg daily, and upadacitinib, 30 mg daily, were associated with reductions in EASI scores; upadacitinib, 15 mg daily, was associated with similar reductions, and tralokinumab, 300 mg every other week, and baricitinib, 2 and 4 mg daily, were associated with fewer reductions.

**Meaning:**

Compared with dupilumab, different doses of abrocitinib, baricitinib, tralokinumab, and upadacitinib were associated with reductions in EASI scores for patients with atopic dermatitis; the relative effect estimates reported herein may help clinicians and patients make informed treatment choices.

## Teaser

This living systematic review and network meta-analysis examines the association of systemic treatments for atopic dermatitis with scores on the Eczema Area and Severity Index, Patient Oriented Eczema Measure, Dermatology Life Quality Index, and Peak Pruritus Numeric Rating Scales.

## Introduction

Systemic immunomodulatory agents used to treat atopic dermatitis include cyclosporine, methotrexate, azathioprine, mycophenolate, and dupilumab, with abrocitinib, baricitinib, tralokinumab, and upadacitinib recently approved in various jurisdictions. The sparsity of head-to-head trials for these medications makes direct comparisons of efficacy and safety difficult. Our previously published network meta-analysis found that dupilumab may have similar efficacy to higher-dose cyclosporine and was superior to methotrexate and azathioprine.^1^ We also found that a number of other investigational medications, including biologics and Janus kinase (JAK) inhibitors showed promise, but had insufficient data to draw firm conclusions about their relative efficacy. Since the publication of the initial review, new randomized clinical trials have been published, and we have continued to update our living systematic review and network meta-analysis.

The objective of this updated systematic review and network meta-analysis is to assess the association of systemic immunomodulatory therapies for moderate-to-severe atopic dermatitis with outcomes, including change in clinical signs, patient-reported symptoms, skin-specific health-related quality of life, and itch severity, prioritizing peak pruritus numeric rating scales.^2^

## Methods

### Search Strategy and Selection Criteria

We search for new studies in the Cochrane Central Register of Controlled Trials (CENTRAL); MEDLINE via Ovid (from 1946); Embase via Ovid (from 1974); Latin American and Caribbean Health Science Information database (LILACS) (from 1982); and the Global Resource of EczemA Trials (GREAT) database, the ISRCTN registry; ClinicalTrials.gov; the Australian New Zealand Clinical Trials Registry; the World Health Organization International Clinical Trials Registry Platform (ICTRP), and the EU Clinical Trials Register for new citations every 4 months, with our last search on June 15, 2021. The ClinicalTrials.gov search functions enable us to search for new and updated entries; other trial registry search updates are only for new entries. The full search strategy can be found at http://eczematherapies.com/research. For published trials, we searched for supplemental results on trial registries and contact study authors and pharmaceutical companies to obtain further data when needed.

### Eligibility Criteria

Complete eligibility criteria have been published previously.^1,3^ We include studies with children and adults with moderate-to-severe AD treated for 8 weeks or longer and at least 2 doses of systemic immunomodulatory therapies with any comparator. We included trials regardless of whether they allowed or did not allow adjunctive topical anti-inflammatory therapy (eg, corticosteroids, calcineurin inhibitors, phosphodiesterase inhibitors). Outcomes were change in investigator-reported clinical signs, prioritizing the EASI (minimal clinically important difference [MCID], 6.6)^4,5^; patient-reported symptoms (MCID 3.4)^5,6^; DLQI (MCID 3.3)^7,8^; and PP-NRS (MCID 2.6).^2^ Prioritization of these outcome measures is aligned with the Harmonizing Outcome Measures for Eczema initiative.^9^ Safety outcomes were withdrawal owing to adverse events; and occurrence of serious adverse events.

### Screening and Abstraction Process

We screened titles, abstracts, and full texts using Covidence software (www.covidence.org/) and performed data abstraction and risk of bias assessment independently in duplicate (for this update, A.M.D. and D.E.M.). Any title or abstract identified by either of the screeners as potentially relevant was advanced to full text review and any discrepancies at full-text screening were resolved by discussion between the two screeners and, when necessary, a senior investigator (C.F.).

For each outcome, we extracted data for short-term (8-16 weeks) and long-term (>16 weeks) time points. Within each short- and long-term period, we extracted outcome data at the latest reported end point during active treatment with initial randomization maintained. Active treatment duration was defined as the time from baseline to the last administered dose plus the interval between doses (eg, if a medication was given every 4 weeks and the last dose was given at 12 weeks, the active treatment duration was considered 16 weeks). For studies where safety outcomes were reported only after an off-treatment monitoring period, we assigned those results to the active treatment timeframe so that our analysis reflects the treatment exposure (eg, for 16 weeks of active treatment with safety follow-up reported at 24 weeks, we considered that safety data to pertain to 16 weeks of treatment). We emailed authors to try to obtain missing outcomes data.

For outcomes, relevant data included the mean change from baseline and a measure of variance. If data were not reported as change from baseline, we used the mean baseline and mean follow-up *P* values. When mean percentage change from baseline was reported, we converted the data to mean change from baseline if baseline values were provided, assuming equal variances at baseline and follow-up and correlation between baseline and follow-up of 0.5.^10^ For withdrawals and serious adverse events, we extracted the number of individuals experiencing the event and the number included in the analysis. If results were only available in figures with exact values not given, we use Engauge Digitizer software (version 10.11) to estimate the values^11^; 2 reviewers (A.M.D. and D.E.M.) derived estimates independently, and we used the mean of their estimates.

### Data Analysis

We performed random-effects network meta-analysis for each outcome within a Bayesian framework using tR statistical software (*gemtc* package. R Foundation) as detailed previously.^1,12^ We used noninformative prior distributions for model parameters given current uncertainty of the relative effectiveness of the treatments.^13^

Within each outcome domain (eg, signs), we analyzed each scale (eg, EASI) separately but did combine different itch numeric rating scales if they assessed worst or peak (as opposed to average) itch. In separate analyses, we combined different scales within each outcome domain using standardized mean differences (SMD).

For withdrawals and serious adverse events, we conducted analyses using a more informative log-normal prior for the heterogeneity parameter.^14^ Specifically, we assumed a reasonable bound would capture the treatment effects, as in the context of pharmaceutical interventions, it is very unlikely that an OR would be greater than 30 or less than 1/30.^15^ Hence, we used a normal prior on the log odds ratios (ORs) such that the 95% coverage included log(1/30) to log(30).

We pooled studies of 8 to 16 weeks of treatment separately from studies longer than 16 weeks. We separated trials with adults from trials with children but included 2 trials of abrocitinib vs placebo and 3 trials of upadacitinib vs placebo that included adolescent and adult participants aged 12 years and older in the adult analysis.^16,17,18^

In our main analyses, dosing regimens were treated as their own network nodes (ie, we did not pool results for different doses of medications). In SMD analyses, to incorporate more information on older medications, we combined different dosing regimens of azathioprine (1-2.5 mg/kg/d),^19,20,21^ methotrexate (10-22.5 mg/week),^20,22^ and cyclosporine lower-dose (150 mg/d, ≤3 mg/kg/d)^22,23,24^ and higher-dose (300 mg/d, 4-5 mg/kg/d).^25,26,27,28^ This is a change from our original analyses when we conducted a secondary analysis separately limited to these combined medication doses and dupilumab. Our current approach allowed us to use all information in the network.

We generated network plots for each analysis. Summary results are presented as mean difference (MD), SMD, or odds ratio with 95% CrI. Where possible, we assessed coherence using node-splitting to compare direct and indirect evidence.^29^ We addressed between-study differences (heterogeneity) by using random effects models and multiple sensitivity and subgroup analyses exploring the effect of important trial characteristics. We summarized treatment rankings using Surface Under the Cumulative Ranking (SUCRA).^30^ We calculated effect estimates for all pairwise comparisons in each network but for clinical relevance we presented effect estimates in this iteration of our study only for placebo and medications currently used in clinical practice or expected to be available soon: azathioprine, methotrexate, cyclosporine, dupilumab, abrocitinib, baricitinib, tralokinumab, upadacitinib, and placebo. League tables for the complete networks are available from the authors on request.

### Subgroup and Sensitivity Analyses

We conducted subgroup analyses separating trials that allow and those that do not allow concomitant topical anti-inflammatory therapy. Studies where patients used topical medications as “rescue” therapy only were categorized as not allowing topical therapy. We conducted sensitivity analyses including only trials with low risk of bias (no items scored as unclear or high risk of bias).

### Risk of Bias and Certainty of Evidence

We assessed risk of bias in individual studies using the Cochrane Risk of Bias tool.^31^ We assessed the overall quality of evidence for each outcome for comparisons between clinically relevant medications using Grading of Recommendations Assessment, Development and Evaluation (GRADE) criteria for network meta-analysis.^32,33^ To create informative GRADE results statements^34^ for treatments currently in use or likely to be approved soon, we defined “little or no difference,” a trivial or small unimportant effect, as being within 1 point on each of the outcome measures or SMD of less than 0.2; “reduces slightly,” a small effect of uncertain importance, as greater than 1 point but less than the MCID or SMD of 0.2-0.8; “reduces,” a moderate effect, greater than the MCID or SMD greater than 0.8; and “results in a large reduction,” a large effect, more than twice the MCID (no equivalent for SMD). For GRADE imprecision assessments, we used the statistical line of no difference to downgrade for point estimates showing a slight or greater difference and used the MCID or SMD of 0.8 to downgrade point estimates showing little or no difference. This is a change from our baseline publication, which used MCIDs as binary cutoffs of clinical significance and used the MCID alone for imprecision estimates.

### Protocol and Updates

Our original protocol was registered on PROSPERO (CRD42018088112) and published.^3^ The living systematic review is updated at www.eczematherapies.com/research. We used the Preferred Reporting Items for Systematic Reviews and Meta-analyses (PRISMA) reporting guidelines extension for network meta-analysis for reporting.^35^ Because this is a systematic review of existing literature, the study was exempt from institutional review board approval and written informed consent.

## Results

**Figure 1.**
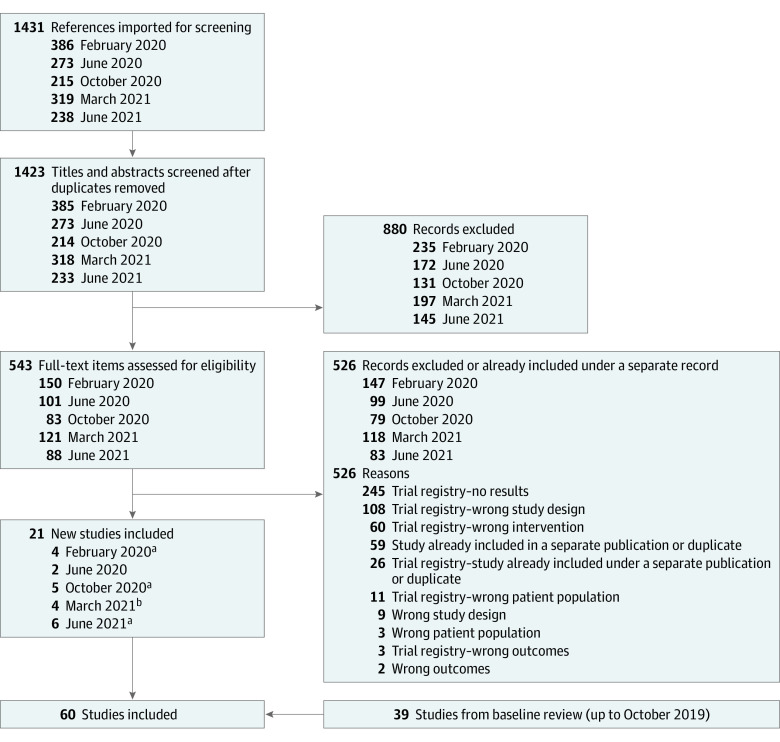
Study Selection Flow Diagram ^a^Two studies included in a single reference. ^b^One study identified through other sources.

Since our baseline review, we screened 1431 titles, abstracts, and trial registry entries, and added 21 new trials for a total of 60 trials with 16 579 patients (Figure 1). New studies included placebo-controlled trials of omalizumab in children (1 trial),^36^ abrocitinib in patients aged 12 to 17 years (1 trial),^37^ 12 years and older (2 trials), and in adults (1 trial that also had an active dupilumab arm),^16,38,39^ baricitinib in adults (5 trials),^40,41,42,43^ dupilumab in children (1 trial)^44^ and adults (1 trial),^45^ lebrikizumab in adults (1 trial),^46^ nemolizumab in adults (1 trial),^47^ tralokinumab in adults (4 trials),^48,49,50^ and upadacitinib in patients aged 12 years and older (3 trials).^17,18^ Most new studies had low risk of bias for most or all elements assessed but 1 study had unclear risk of bias from selective reporting, 4 studies from random sequence generation, and 8 from incomplete outcome data; 1 study had high risk of bias for incomplete outcome data (eTable 1 in the Supplement). Complete study characteristics and extracted outcomes data are found at http://eczematherapies.com/research.

**Figure 2.**
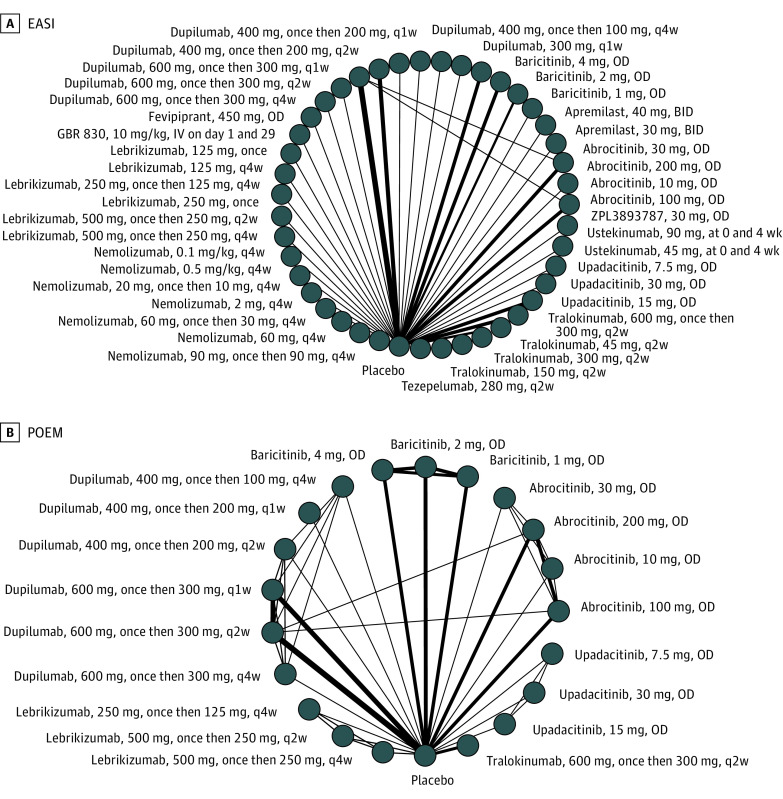
Network Graphs of Studies Included in the Analysis of Adults Treated Between 8 and 16 Weeks Change in (A) Eczema Area and Severity Index (EASI), (B) Patient Oriented Eczema Measure (POEM). The width of each line connecting 2 treatments (nodes) is proportional to the number of head-to-head trials for that comparison. BID indicates twice per day; OD, once daily; q1w, once weekly; q2w, every 2 weeks; q4w, every 4 weeks.

**Figure 3.**
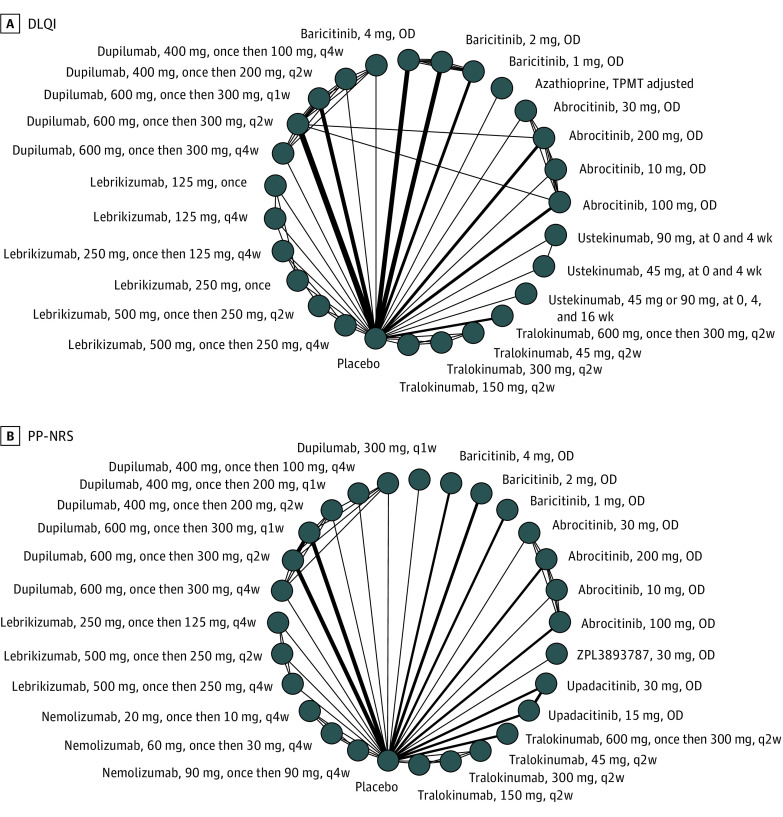
Network Graphs of Studies Included in the Analysis of Adults Treated Between 8 and 16 Weeks A, Dermatology Life Quality Index (DLQI), and (B) Peak Pruritus Numeric Rating Scales (PP-NRS). The width of each line connecting 2 treatments (nodes) is proportional to the number of head-to-head trials for that comparison. OD indicates once daily; q1w, once weekly; q2w, every 2 weeks; q4w, every 4 weeks.

There is insufficient data to conduct network meta-analysis for long-term outcomes or trials of children. Our network graphs for adults treated for up to 16 weeks still show a predominantly “spoke-and-wheel” distribution with various medication doses connected to placebo and different doses of the same medication connected to each other (Figure 2 and Figure 3). There were few connections between nodes of different active medications.

**Figure 4.**
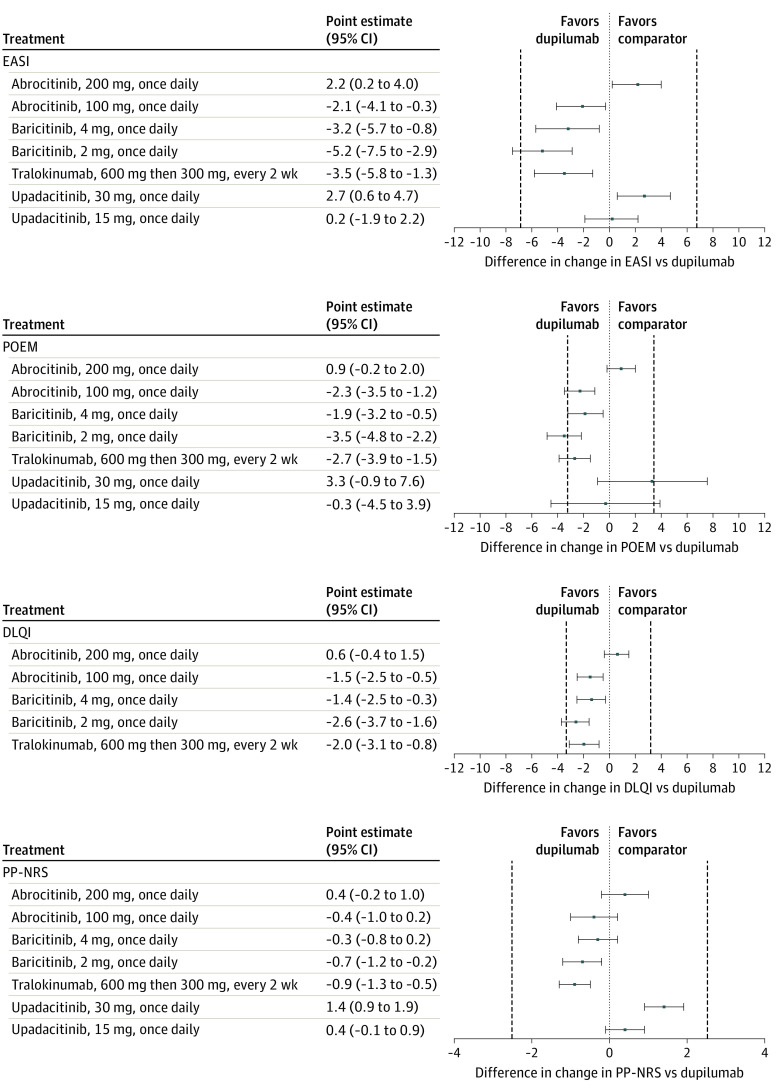
Forest Plots of Network Meta-analysis Results of Adults Treated Between 8 and 16 Weeks Forest plots showing change in Eczema Area and Severity Index (EASI), Patient Oriented Eczema Measure (POEM), Dermatology Life Quality Index (DLQI), and Peak Pruritus Numeric Rating Scales (PP-NRS). Results are presented for medications currently used in clinical practice or likely to be approved soon vs dupilumab on-label dosing (600 mg then 300 mg every 2 weeks). Results are presented as mean difference (MD) with 95% credible intervals (CrI). Positive values represent improvement in the disease state vs dupilumab. The vertical dashed lines represent the minimal clinically important difference for each scale. Authors can be contacted for relative effect estimates for all pairwise comparisons in the networks.

Up to 16 weeks of treatment in adults, abrocitinib, 200 mg daily (MD, 2.2; 95% CrI, 0.2-4.0; high certainty) and upadacitinib, 30 mg daily (MD, 2.7; 95% CrI, 0.6-4.7; high certainty) were associated with reduced EASI scores slightly more than dupilumab, 600 mg then 300 mg every 2 weeks. Abrocitinib, 100 mg daily (MD, −2.1; 95% CrI, −4.1 to −0.3; high certainty), baricitinib, 4 mg daily (MD, −3.2; 95% CrI, −5.7 to −0.8; high certainty), baricitinib, 2 mg daily (MD, −5.2; 95% CrI, −7.5 to −2.9; high certainty), and tralokinumab, 600 mg then 300 mg every 2 weeks (MD, −3.5; 95% CrI, −5.8 to −1.3; high certainty) reduced EASI slightly less than dupilumab and there was little or no difference between upadacitinib, 15 mg daily, and dupilumab (MD, 0.2; 95% CrI, −1.9 to 2.2; high certainty). The pattern of results was similar for change in POEM (Figure 4; eTables 4 and 5 in the Supplement), DLQI (Figure 4; eTables 6 and 7 in the Supplement), and PP-NRS (Figure 4; eTable 8 and 9 in the Supplement).

In SMD analyses, the relative outcomes of conventional systemic agents vs dupilumab were similar to our baseline network meta-analyses (eFigures 1, 2, and 3 and eTables 10, 11, 12, 13, 14, and 15 in the Supplement).^1^ Higher-dose cyclosporine was associated with improved clinical signs slightly better than dupilumab (SMD, −0.2; 95% CrI, −0.8 to 0.5; low certainty). Lower-dose cyclosporine (SMD, 0.2; 95% CrI, −0.5 to 0.8; low certainty), methotrexate (SMD, 0.2; 95% CrI, −0.4 to 0.9; low certainty), and azathioprine (SMD, 0.3; 95% CrI, −0.1 to 0.7; low certainty) were associated with reduced signs slightly less than dupilumab, but certainty of evidence was low owing to concerns related to risk of bias of included trials and imprecision reflected in wide credible intervals.

Subgroup analyses limited to trials with and without concomitant use of topical anti-inflammatory treatment, respectively, did not substantially alter effect estimates or conclusions.

For withdrawal owing to adverse events among patients receiving abrocitinib, baricitinib, dupilumab, tralokinumab, upadacitinib, and placebo, credible intervals were wide, contributing to lower certainty evidence, so we were unable to make clinically useful conclusions (eFigure 4, and eTables 16 and 17 in the Supplement). Abrocitinib, 100 mg daily, was associated with more serious adverse events than dupilumab (OR, 2.6; 95% CrI, 1.1-6.4; low certainty) and dupilumab was associated with fewer serious adverse events than placebo (OR, 0.5; 95% CrI, 0.3-0.8; moderate certainty) (eFigure 5, and eTables 18 and 19 in the Supplement).

Node-splitting showed no evidence of incoherence in the primary outcomes analyses. For withdrawals and serious adverse events, there was evidence of incoherence for estimates between 2 different doses of dupilumab and between 2 different doses of baricitinib, likely owing to low adverse event rates leading to imprecise and unstable estimates.

Results were consistent in sensitivity analyses, but as trials were removed, so were some nodes, and effect estimates became less precise.

## Discussion

In this updated network meta-analysis including multiple new phase 3 clinical trials, dupilumab, abrocitinib, baricitinib, tralokinumab, and upadacitinib was associated with improved index scores in adults with moderate-to-severe atopic dermatitis treated for up to 16 weeks. There was a consistent pattern across our main outcomes (EASI, POEM, DLQI, PP-NRS), with abrocitinib, 200 mg daily, and upadacitinib, 30 mg daily, associated with slightly better index scores, upadacitinib, 15 mg daily, associated with similar index scores, and abrocitinib, 100 mg daily, baricitinib, 2 and 4 mg daily, and tralokinumab, 600 mg then 300 mg every 2 weeks, associated with slightly worse index scores compared with dupilumab. Most effect estimates were within the range of the MCID for each outcome scale. There were no new data for azathioprine, cyclosporine, or methotrexate; as in our baseline SMD analysis, higher-dose cyclosporine may have shown similar outcomes, and methotrexate and azathioprine were associated with worse results than dupilumab, but with imprecision around the effect estimates.

This update includes the first trial with head-to-head data comparing an oral JAK inhibitor (abrocitinib) against dupilumab.^51^ Results from that trial were consistent with our indirect network meta-analysis findings. After our latest search, a trial directly comparing upadacitinib and dupilumab was published; they presented mostly binary outcomes so the results of that trial were not directly comparable with our analysis, but the trial also found upadacitinib, 30 mg daily, to be more effective than dupilumab.^52^

We found that abrocitinib, 100 mg daily, was associated with 2.6 times the odds of serious adverse events compared with dupilumab, but the effect estimate was lower (1.4) with credible intervals overlapping the null for abrocitinib, 200 mg daily, compared with dupilumab. We found lower rates of serious adverse events for dupilumab compared with placebo, which is surprising as one would not expect serious adverse events related to placebo. In a network meta-analysis of psoriasis treatments, psoriasis flares coded as adverse events led to similar spurious findings.^53^ However, targeted treatment may lead to decreased atopic dermatitis-associated infections such as bacterial skin infections and eczema herpeticum,^54^ which could also explain placebo having higher adverse event rates in our analysis. Although these findings warrant further study, generally low rates of withdrawals owing to adverse events and serious adverse events in the included studies make our estimates unstable and preclude strong conclusions on relative safety. Observational studies, including those with large sample sizes and longer-term follow-up, are needed to detect any important but uncommon serious adverse events.

Our results for dupilumab and tralokinumab were consistent with a previous Cochrane network meta-analyses of systemic treatments for eczema^55^ and our finding that abrocitinib, upadacitinib, and dupilumab were among the most effective treatments is consistent with a recent industry-sponsored network meta-analyses.^56^ However, there are some important differences between those studies and this one, including that other network meta-analyses assess mainly dichotomous outcomes (eg, proportion of patients with 75% improvement in EASI), whereas we assessed change in continuous outcome scales. Though responder analyses are problematic,^57^ and dichotomous outcomes fail to capture meaningful improvement for some atopic dermatitis patients,^58^ they are commonly used as primary and key secondary end points in atopic dermatitis clinical trials, so we plan to expand our outcomes to include them in future updates. The Cochrane review pooled results across doses of systemic medications in their primary analysis; although this increases statistical power, it sacrifices accuracy. We treated each dose separately because some medications, including abrocitinib, baricitinib, and upadacitinib demonstrate a clear dose-response effect and will likely not be used at lower doses assessed only in early-phase clinical trials (eg, abrocitinib, 10 mg daily).

### Limitations

This analysis was limited by differences in the design of included trials, but inclusion criteria for newly added studies are similar, suggesting that trials are becoming more standardized as the field develops. We pooled trials with durations between 8 and 16 weeks; this may have favored medications in 16-week trials, with more time to improve atopic dermatitis. An important variable in trial design is whether concomitant topical anti-inflammatory therapies are permitted along with the systemic medications under study. Topical medications may increase both placebo and active arm treatment responses, and concomitant topical therapy is likely more reflective of actual practice patterns.^59^ In our analyses limited to studies permitting and not permitting these agents, respectively, relative effect estimates did not change substantially. Our networks remain sparse for medications other than dupilumab, abrocitinib, baricitinib, tralokinumab, and upadacitinib. Our results have limited generalizability to populations underrepresented in clinical trials, including older patients and patients with comorbidities,^60^ and we are as yet unable to conduct network meta-analyses for long-term outcomes and studies of children owing to insufficient trial data examining these populations.

## Conclusions

In our updated living systematic review and network meta-analysis of systemic treatments for atopic dermatitis, the JAK inhibitors abrocitinib, baricitinib, and upadacitinib and the biologic tralokinumab were associated with improved index scores compared with placebo in randomized clinical trials and were associated with comparable improvements in index scores to dupilumab. Our finding that abrocitinib, 200 mg daily, and upadacitinib, 30 mg daily, may be associated with slightly better index scores than dupilumab, 600 mg then 300 mg every 2 weeks, is supported by head-to-head trials.^51,52^ Our results may aid shared decision-making between clinicians and patients seeking to understand the relative merits of different treatment options for moderate-to-severe atopic dermatitis.

## Notes

Supplement. eTable 1. Risk of bias assessment for new studies added since baseline review eTable 2. League table for change in EASI eTable 3. GRADE certainty ratings for change in EASI 5 eTable 4. League table for change in POEM eTable 5. GRADE certainty ratings for change in POEM eTable 6. League table for change in DLQI eTable 7. GRADE certainty ratings for change in DLQI eTable 8. League table for change in PP-NRS eTable 9. GRADE certainty ratings for change in PP-NRS eFigure 1. Network graphs for change in signs on the standardized mean difference scale eTable 10. League table for change in signs on the standardized mean difference scale eTable 11. GRADE certainty ratings for change in signs on the standardized mean difference scale eFigure 2. Network graphs of change in QoL on the standardized mean difference scale eTable 12. League table for change in QoL on the standardized mean difference scale eTable 13. GRADE certainty ratings for change in QoL on the standardized mean difference scale eFigure 3. Network graphs of change in itch on the standardized mena difference scale eTable 14. League table for change in itch on the standardized mean difference scale eTable 15. GRADE certainty ratings for change in itch on the standardized mean difference scale eFigure 4. Network graphs for withdrawal due to adverse events eTable 16. League table for withdrawal due to adverse events eTable 17. GRADE certainty ratings for withdrawal due to adverse events eFigure 5. Network graphs for serious adverse events eTable 18. League table for serious adverse events eTable 19. GRADE certainty ratings for serious adverse events eReferences
