# Supplementary material for: Systemic Immunomodulatory Treatments for Atopic Dermatitis: Update of a Living Systematic Review and Network Meta-analysis
Source: JAMA Dermatol. 2022 Mar 16;158(5):523–32. doi: 10.1001/jamadermatol.2022.0455 (PMC8928094; doi:10.1001/jamadermatol.2022.0455)
Supplement: Supplement. — eTable 1. Risk of bias assessment for new studies added since baseline review eTable 2. League table for change in EASI eTable 3. GRADE certainty ratings for change in EASI 5 eTable 4. League table for change in POEM eTable 5. GRADE certainty ratings for change in POEM eTable 6. League table for change in DLQI eTable 7. GRADE certainty ratings for change in DLQI eTable 8. League table for change in PP-NRS eTable 9. GRADE certainty ratings for change in PP-NRS eFigure 1. Network graphs for change in signs on the standardized mean difference scale eTable 10. League table for change in signs on the standardized mean difference scale eTable 11. GRADE certainty ratings for change in signs on the standardized mean difference scale eFigure 2. Network graphs of change in QoL on the standardized mean difference scale eTable 12. League table for change in QoL on the standardized mean difference scale eTable 13. GRADE certainty ratings for change in QoL on the standardized mean difference scale eFigure 3. Network graphs of change in itch on the standardized mena difference scale eTable 14. League table for change in itch on the standardized mean difference scale eTable 15. GRADE certainty ratings for change in itch on the standardized mean difference scale eFigure 4. Network graphs for withdrawal due to adverse events eTable 16. League table for withdrawal due to adverse events eTable 17. GRADE certainty ratings for withdrawal due to adverse events eFigure 5. Network graphs for serious adverse events eTable 18. League table for serious adverse events eTable 19. GRADE certainty ratings for serious adverse events eReferences [file jamadermatol-e220455-s001.pdf]

Supplemental Online Content

Drucker AM, Morra DE, Prieto-Merino D, et al. Systemic immunomodulatory treatments for atopic dermatitis: update of a living systematic review and network meta-analysis. *JAMA Dermatol*. March 16, 2022. doi:10.1001/jamadermatol.2022.0455

eTable 1. Risk of bias assessment for new studies added since baseline review
eTable 2. League table for change in EASI
eTable 3. GRADE certainty ratings for change in EASI
eTable 4. League table for change in POEM
eTable 5. GRADE certainty ratings for change in POEM
eTable 6. League table for change in DLQI
eTable 7. GRADE certainty ratings for change in DLQI
eTable 8. League table for change in PP-NRS
eTable 9. GRADE certainty ratings for change in PP-NRS
eFigure 1. Network graphs for change in signs on the standardized mean difference scale
eTable 10. League table for change in signs on the standardized mean difference scale
eTable 11. GRADE certainty ratings for change in signs on the standardized mean difference scale
eFigure 2. Network graphs of change in QoL on the standardized mean difference scale
eTable 12. League table for change in QoL on the standardized mean difference scale
eTable 13. GRADE certainty ratings for change in QoL on the standardized mean difference scale
eFigure 3. Network graphs of change in itch on the standardized mena difference scale
eTable 14. League table for change in itch on the standardized mean difference scale
eTable 15. GRADE certainty ratings for change in itch on the standardized mean difference scale
eFigure 4. Network graphs for withdrawal due to adverse events
eTable 16. League table for withdrawal due to adverse events
eTable 17. GRADE certainty ratings for withdrawal due to adverse events
eFigure 5. Network graphs for serious adverse events
eTable 18. League table for serious adverse events
eTable 19. GRADE certainty ratings for serious adverse events
eReferences

This supplemental material has been provided by the authors to give readers additional information about their work.

© 2022 Drucker AM, et al. *JAMA Dermatology*.

© 2022 Drucker AM, et al. *JAMA Dermatology*.

eTable 1. Risk of bias assessment for new studies added since baseline review

	Random sequence generation	Allocation concealment	Blinding of participants and personnel	Blinding of outcome assessment	Incomplete outcome data	Selective reporting	Other bias
Chan UK 2020[1]	Low	Low	Low	Low	Low	Low	Low
Simpson 2020 BREEZE-AD 1[2]	Unclear	Low	Low	Low	Unclear	Low	Low
Simpson 2020 BREEZE-AD 2[2]	Unclear	Low	Low	Low	Unclear	Low	Low
Simpson USA 2020 Abrocitinib/ NCT03349060[3]	Low	Low	Low	Low	Low	Low	Low
Guttman-Yassky USA 2020 Lebrikizumab[4]	Unclear	Low	Low	Low	Unclear	Low	Low
Paller USA 2020 Dupilumab[5]	Low	Low	Low	Low	Low	Low	Low
Reich Germany 2020 (BREEZE-AD7)[6]	Low	Low	Low	Low	Low	Low	Low
Silverberg USA 2020 (ECZTRA3)[7]	Low	Low	Low	Low	Low	Low	Low
Wollenberg Germany 2020 (ECZTRA1)[8]	Low	Low	Low	Low	Low	Low	Low
Wollenberg Germany 2020 (ECZTRA2)[8]	Low	Low	Low	Low	Low	Low	Low
Kabashima Japan 2020[9]	Unclear	Low	Low	Low	Low	Low	Low
Silverberg USA 2020 (JADE MONO-2)[10]	Low	Low	Low	Low	Unclear	Low	Low
Simpson 2021 (BREEZE-AD5)[11]	Low	Low	Low	Low	High	Low	Low
NCT03428100 (BREEZE-AD4)[12]	Low	Low	Low	Low	Unclear	Low	Low
NCT03720470 (JADE Compare)[13]	Low	Low	Low	Low	Low	Low	Low
NCT03912259[14]	Low	Low	Low	Low	Low	Low	Low
NCT03796676 (JADE TEEN)[15]	Low	Low	Low	Low	Low	Low	Low
Guttman USA 2021 (MEASURE UP 1)[16]	Low	Low	Low	Low	Unclear	Low	Low

© 2022 Drucker AM, et al. *JAMA Dermatology*.

Guttman USA 2021 (MEASURE UP 2)[16]	Low	Low	Low	Low	Unclear	Low	Low
Reich Germany 2021 (AD Up)[17]	Low	Low	Low	Low	Unclear	Unclear	Low
Merola USA 2021 (ECZTRA5)[18]	Low	Low	Low	Low	Low	Low	Low

© 2022 Drucker AM, et al. *JAMA Dermatology*.

eTable 2. League table with relative effect estimates for change in EASI up to 16 weeks of treatment in adults for placebo and medications used in clinical practice or likely to be approved soon. Results for other pairwise comparisons in the network can be requested from the authors. Results are presented as change in EASI (95% CrI). A positive effect estimate in a given cell favors the row-defining treatment. A negative effect estimate in a given cell favors the column-defining treatment. The bottom row contains the Surface Under the Cumulative Ranking (SUCRA) value for the column-defining treatment.

	Abrocitinib 100 mg daily	Abrocitinib 200 mg daily	Baricitinib 2 mg daily	Baricitinib 4 mg daily	Dupilumab 600 mg then 300 mg q2 weeks	Placebo	Tralokinumab 600 mg then 300 mg q2 weeks	Upadacitinib 15 mg daily	Upadacitinib 30 mg daily
Abrocitinib 100 mg daily		-4.3 (-6, -2.7)	3.1 (0.5, 5.6)	1.1 (-1.6, 3.7)	-2.1 (-4.1, -0.3)	8.6 (6.9, 10.3)	1.4 (-1.1, 3.9)	-2.3 (-4.7, -0.1)	-4.9 (-7.2, -2.6)
Abrocitinib 200 mg daily	4.3 (2.7, 6)		7.4 (4.8, 9.9)	5.4 (2.7, 8)	2.2 (0.2, 4)	13 (11.3, 14.7)	5.7 (3.2, 8.2)	2 (-0.3, 4.3)	-0.6 (-2.9, 1.7)
Baricitinib 2 mg daily	-3.1 (-5.6, -0.5)	-7.4 (-9.9, -4.8)		-2 (-4.1, 0.1)	-5.2 (-7.5, -2.9)	5.6 (3.7, 7.5)	-1.7 (-4.3, 1)	-5.4 (-7.9, -2.9)	-7.9 (-10.4, -5.5)
Baricitinib 4 mg daily	-1.1 (-3.7, 1.6)	-5.4 (-8, -2.7)	2 (-0.1, 4.1)		-3.2 (-5.7, -0.8)	7.6 (5.5, 9.6)	0.3 (-2.4, 3)	-3.4 (-6, -0.8)	-5.9 (-8.5, -3.4)
Dupilumab 600 mg then 300 mg q2 weeks	2.1 (0.3, 4.1)	-2.2 (-4, -0.2)	5.2 (2.9, 7.5)	3.2 (0.8, 5.7)		10.8 (9.5, 12.2)	3.5 (1.3, 5.8)	-0.2 (-2.2, 1.9)	-2.7 (-4.7, -0.6)
Placebo	-8.6 (-10.3, -6.9)	-13 (-14.7, -11.3)	-5.6 (-7.5, -3.7)	-7.6 (-9.6, -5.5)	-10.8 (-12.2, -9.5)		-7.3 (-9.1, -5.4)	-11 (-12.5, -9.5)	-13.5 (-15.1, -12)
Tralokinumab 600 mg then 300 mg q2 weeks	-1.4 (-3.9, 1.1)	-5.7 (-8.2, -3.2)	1.7 (-1, 4.3)	-0.3 (-3, 2.4)	-3.5 (-5.8, -1.3)	7.3 (5.4, 9.1)		-3.7 (-6.1, -1.4)	-6.3 (-8.6, -3.9)
Upadacitinib 15 mg daily	2.3 (0.1, 4.7)	-2 (-4.3, 0.3)	5.4 (2.9, 7.9)	3.4 (0.8, 6)	0.2 (-1.9, 2.2)	11 (9.5, 12.5)	3.7 (1.4, 6.1)		-2.5 (-4, -1)
Upadacitinib 30 mg daily	4.9 (2.6, 7.2)	0.6 (-1.7, 2.9)	7.9 (5.5, 10.4)	5.9 (3.4, 8.5)	2.7 (0.6, 4.7)	13.5 (12, 15.1)	6.3 (3.9, 8.6)	2.5 (1, 4)	
SUCRA	0.76	0.94	0.55	0.70	0.86	0.11	0.68	0.87	0.95

© 2022 Drucker AM, et al. *JAMA Dermatology*.

eTable 3. GRADE certainty ratings for change in EASI up to 16 weeks of treatment in adults for placebo and medications used in clinical practice or likely to be approved soon. Results are presented as change in EASI (95% CrI). Negative effect estimates favor the treatment listed in the intervention column; positive effect estimates favor the comparator.

Intervention	Comparator	Head-to-head trials, n	Direct estimate (95% CrI); Certainty of Evidence	Indirect estimate (95% CrI); Certainty of Evidence	NMA Estimate (95% CrI); Certainty of Evidence
Abrocitinib 100 mg daily	Abrocitinib 200 mg daily	4	4.3 (2.7, 6); high	--	4.3 (2.7, 6); high
Abrocitinib 100 mg daily	Baricitinib 2 mg daily	0	--	-3.1 (-5.6, -0.5); high	-3.1 (-5.6, -0.5); high
Abrocitinib 100 mg daily	Baricitinib 4 mg daily	0	--	-1.1 (-3.7, 1.6); high	-1.1 (-3.7, 1.6); moderate[a]
Abrocitinib 100 mg daily	Dupilumab	1	0.6 (-1.9, 3.0); high	3.9 (1.4, 6.6); high	2.1 (0.3, 4.1); high
Abrocitinib 100 mg daily	Placebo	4	-8.6 (-10.3, -6.9); high	--	-8.6 (-10.3, -6.9); high
Abrocitinib 100 mg daily	Tralokinumab	0	--	-1.4 (-3.9, 1.1); high	-1.4 (-3.9, 1.1); moderate[a]
Abrocitinib 100 mg daily	Upadacitinib 15 mg daily	0	--	2.3 (0.1, 4.7); high	2.3 (0.1, 4.7); high
Abrocitinib 100 mg daily	Upadacitinib 30 mg daily	0	--	4.9 (2.6, 7.2); high	4.9 (2.6, 7.2); high
Abrocitinib 200 mg daily	Baricitinib 2 mg daily	0	--	-7.4 (-9.9, -4.8); high	-7.4 (-9.9, -4.8); high
Abrocitinib 200 mg daily	Baricitinib 4 mg daily	0	--	-5.4 (-8, -2.7); high	-5.4 (-8, -2.7); high
Abrocitinib 200 mg daily	Dupilumab	1	-3.0 (-5.4, -0.5); high	-1.3 (-3.8, 1.3); high	-2.2 (-4, -0.2); high
Abrocitinib 200 mg daily	Placebo	4	-13 (-14.7, -11.3); high	--	-13 (-14.7, -11.3); high
Abrocitinib 200 mg daily	Tralokinumab	0	--	-5.7 (-8.2, -3.2); high	-5.7 (-8.2, -3.2); high
Abrocitinib 200 mg daily	Upadacitinib 15 mg daily	0	--	-2 (-4.3, 0.3); high	-2 (-4.3, 0.3); moderate[a]
Abrocitinib 200 mg daily	Upadacitinib 30 mg daily	0	--	0.6 (-1.7, 2.9); high	0.6 (-1.7, 2.9); high
Baricitinib 2 mg daily	Baricitinib 4 mg daily	4	2 (-0.1, 4.1); moderate*	--	2 (-0.1, 4.1); low[a]
Baricitinib 2 mg daily	Dupilumab	0	--	5.2 (2.9, 7.5); high	5.2 (2.9, 7.5); high
Baricitinib 2 mg daily	Placebo	5	-5.6 (-7.5, -3.7); high	--	-5.6 (-7.5, -3.7); high
Baricitinib 2 mg daily	Tralokinumab	0	--	1.7 (-1, 4.3); high	1.7 (-1, 4.3); moderate[a]

© 2022 Drucker AM, et al. *JAMA Dermatology*.

Baricitinib 2 mg daily	Upadacitinib 15 mg daily	0	--	5.4 (2.9, 7.9); high	5.4 (2.9, 7.9); high
Baricitinib 2 mg daily	Upadacitinib 30 mg daily	0	--	7.9 (5.5, 10.4); high	7.9 (5.5, 10.4); high
Baricitinib 4 mg daily	Dupilumab	0	--	3.2 (0.8, 5.7); high	3.2 (0.8, 5.7); high
Baricitinib 4 mg daily	Placebo	4	-7.6 (-9.6, -5.5); high	--	-7.6 (-9.6, -5.5); high
Baricitinib 4 mg daily	Tralokinumab	0	--	-0.3 (-3, 2.4); high	-0.3 (-3, 2.4); high
Baricitinib 4 mg daily	Upadacitinib 15 mg daily	0	--	3.4 (0.8, 6); high	3.4 (0.8, 6); high
Baricitinib 4 mg daily	Upadacitinib 30 mg daily	0	--	5.9 (3.4, 8.5); high	5.9 (3.4, 8.5); high
Dupilumab	Placebo	7	-10.8 (-12.2, -9.5); high	--	-10.8 (-12.2, -9.5); high
Dupilumab	Tralokinumab	0	--	-3.5 (-5.8, -1.3); high	-3.5 (-5.8, -1.3); high
Dupilumab	Upadacitinib 15 mg daily	0	--	0.2 (-1.9, 2.2); high	0.2 (-1.9, 2.2); high
Dupilumab	Upadacitinib 30 mg daily	0	--	2.7 (0.6, 4.7); high	2.7 (0.6, 4.7); high
Tralokinumab	Upadacitinib 15 mg daily	0	--	3.7 (1.4, 6.1); high	3.7 (1.4, 6.1); high
Tralokinumab	Upadacitinib 30 mg daily	0	--	6.3 (3.9, 8.6); high	6.3 (3.9, 8.6); high
Tralokinumab	Placebo	3	-7.3 (-9.1, -5.4); high	--	-7.3 (-9.1, -5.4); high
Upadacitinib 15 mg daily	Upadacitinib 30 mg daily	4	2.5 (1, 4); high	--	2.5 (1, 4); high
Upadacitinib 15 mg daily	Placebo		-11 (-12.5, -9.5); high	--	-11 (-12.5, -9.5); high
Upadacitinib 30 mg daily	Placebo		-13.5 (-15.1, -12); high	--	-13.5 (-15.1, -12); high

Dose of dupilumab and tralokinumab is 600 mg then 300 mg every 2 weeks

The network meta-analysis certainty ratings start at the higher of the direct or indirect ratings and can then be rated down further for incoherence (based on the results of node splitting analyses) and imprecision.

* Rated down for inconsistency
a. Rated down for imprecision

© 2022 Drucker AM, et al. *JAMA Dermatology*.

eTable 4. League table with relative effect estimates for change in POEM up to 16 weeks of treatment in adults for placebo and medications used in clinical practice or likely to be approved soon. Results for other pairwise comparisons in the network can be requested from the authors. Results are presented as change in POEM (95% CrI). A positive effect estimate in a given cell favors the row-defining treatment. A negative effect estimate in a given cell favors the column-defining treatment. The bottom row contains the Surface Under the Cumulative Ranking (SUCRA) value for the column-defining treatment.

	Abrocitinib 100 mg daily	Abrocitinib 200 mg daily	Baricitinib 2 mg daily	Baricitinib 4 mg daily	Dupilumab 600 mg then 300 mg q2 weeks	Placebo	Tralokinumab 600 mg then 300 mg q2 weeks	Upadacitinib 15 mg daily	Upadacitinib 30 mg daily
Abrocitinib 100 mg daily		-3.2 (-4.2, -2.2)	1.2 (-0.4, 2.7)	-0.5 (-2.1, 1.1)	-2.3 (-3.5, -1.2)	5 (3.9, 6)	0.4 (-1.1, 1.8)	-2 (-6.3, 2.2)	-5.6 (-10, -1.5)
Abrocitinib 200 mg daily	3.2 (2.2, 4.2)		4.4 (2.9, 5.9)	2.7 (1.2, 4.3)	0.9 (-0.2, 2)	8.2 (7.1, 9.2)	3.6 (2.1, 5)	1.2 (-3, 5.4)	-2.4 (-6.7, 1.7)
Baricitinib 2 mg daily	-1.2 (-2.7, 0.4)	-4.4 (-5.9, -2.9)		-1.6 (-2.8, -0.5)	-3.5 (-4.8, -2.2)	3.8 (2.6, 4.9)	-0.8 (-2.3, 0.7)	-3.2 (-7.5, 1.1)	-6.8 (-11.1, -2.6)
Baricitinib 4 mg daily	0.5 (-1.1, 2.1)	-2.7 (-4.3, -1.2)	1.6 (0.5, 2.8)		-1.9 (-3.2, -0.5)	5.4 (4.2, 6.6)	0.8 (-0.7, 2.4)	-1.5 (-5.8, 2.8)	-5.2 (-9.5, -1)
Dupilumab 600 mg then 300 mg q2 weeks	2.3 (1.2, 3.5)	-0.9 (-2, 0.2)	3.5 (2.2, 4.8)	1.9 (0.5, 3.2)		7.3 (6.6, 8)	2.7 (1.5, 3.9)	0.3 (-3.9, 4.5)	-3.3 (-7.6, 0.8)
Placebo	-5 (-6, -3.9)	-8.2 (-9.2, -7.1)	-3.8 (-4.9, -2.6)	-5.4 (-6.6, -4.2)	-7.3 (-8, -6.6)		-4.6 (-5.6, -3.6)	-7 (-11.1, -2.9)	-10.6 (-14.8, -6.6)
Tralokinumab 600 mg then 300 mg q2 weeks	-0.4 (-1.8, 1.1)	-3.6 (-5, -2.1)	0.8 (-0.7, 2.3)	-0.8 (-2.4, 0.7)	-2.7 (-3.9, -1.5)	4.6 (3.6, 5.6)		-2.4 (-6.6, 1.8)	-6 (-10.3, -1.9)
Upadacitinib 15 mg daily	2 (-2.2, 6.3)	-1.2 (-5.4, 3)	3.2 (-1.1, 7.5)	1.5 (-2.8, 5.8)	-0.3 (-4.5, 3.9)	7 (2.9, 11.1)	2.4 (-1.8, 6.6)		-3.7 (-7.7, 0.4)
Upadacitinib 30 mg daily	5.6 (1.5, 10)	2.4 (-1.7, 6.7)	6.8 (2.6, 11.1)	5.2 (1, 9.5)	3.3 (-0.8, 7.6)	10.6 (6.6, 14.8)	6 (1.9, 10.3)	3.7 (-0.4, 7.7)	
SUCRA	0.49	0.85	0.36	0.54	0.72	0.13	0.44	0.69	0.95

© 2022 Drucker AM, et al. *JAMA Dermatology*.

eTable 5. GRADE certainty ratings for change in POEM up to 16 weeks of treatment in adults for placebo and medications used in clinical practice or likely to be approved soon. Results are presented as change in EASI (95% CrI). Negative effect estimates favor the treatment listed in the intervention column; positive effect estimates favor the comparator.

Intervention	Comparator	Head-to-head trials, n	Direct estimate (95% CrI); Certainty of Evidence	Indirect estimate (95% CrI); Certainty of Evidence	NMA Estimate (95% CrI); Certainty of Evidence
Abrocitinib 100 mg daily	Abrocitinib 200 mg daily	4	3.2 (2.2, 4.2); high	--	3.2 (2.2, 4.2); high
Abrocitinib 100 mg daily	Baricitinib 2 mg daily	0	--		-1.2 (-2.7, 0.4); moderate[a]
Abrocitinib 100 mg daily	Baricitinib 4 mg daily	0	--		0.5 (-1.1, 2.1); high
Abrocitinib 100 mg daily	Dupilumab	1	1.6 (0.0, 3.2); high	2.9 (1.3, 4.5); high	2.3 (1.2, 3.5); high
Abrocitinib 100 mg daily	Placebo	4	-5 (-6, -3.9); high	--	-5 (-6.0, -3.9); high
Abrocitinib 100 mg daily	Tralokinumab	0	--	-0.4 (-1.8, 1.1); high	-0.4 (-1.8, 1.1); high
Abrocitinib 100 mg daily	Upadacitinib 15 mg daily	0	--	2 (-2.2, 6.3); high	2 (-2.2, 6.3); moderate[a]
Abrocitinib 100 mg daily	Upadacitinib 30 mg daily	0	--	5.6 (1.5, 10); high	5.6 (1.5, 10); high
Abrocitinib 200 mg daily	Baricitinib 2 mg daily	0	--	-4.4 (-5.9, -2.9); high	-4.4 (-5.9, -2.9); high
Abrocitinib 200 mg daily	Baricitinib 4 mg daily	0	--	-2.7 (-4.3, -1.2); high	-2.7 (-4.3, -1.2); high
Abrocitinib 200 mg daily	Dupilumab	1	-1.7 (-3.3, -0.1); high	-0.2 (-1.7, 1.3); high	-0.9 (-2.0, 0.2); high
Abrocitinib 200 mg daily	Placebo	4	-8.2 (-9.2, -7.1); high	--	-8.2 (-9.2, -7.1); high
Abrocitinib 200 mg daily	Tralokinumab	0	--	-3.6 (-5, -2.1); high	-3.6 (-5, -2.1); high
Abrocitinib 200 mg daily	Upadacitinib 15 mg daily	0	--	-1.2 (-5.4, 3); high	-1.2 (-5.4, 3.0); moderate[a]
Abrocitinib 200 mg daily	Upadacitinib 30 mg daily	0	--	2.4 (-1.7, 6.7); high	2.4 (-1.7, 6.7); moderate[a]
Baricitinib 2 mg daily	Baricitinib 4 mg daily	4	1.6 (0.5, 2.8); high	--	1.6 (0.5, 2.8); high
Baricitinib 2 mg daily	Dupilumab	0	--	3.5 (2.2, 4.8); high	3.5 (2.2, 4.8); high
Baricitinib 2 mg daily	Placebo	5	-3.8 (-4.9, -2.6); high	--	-3.8 (-4.9, -2.6); high
Baricitinib 2 mg daily	Tralokinumab	0	--	0.8 (-0.7, 2.3); high	0.8 (-0.7, 2.3); high
Baricitinib 2 mg daily	Upadacitinib 15 mg daily	0	--	3.2 (-1.1, 7.5); high	3.2 (-1.1, 7.5); moderate[a]
Baricitinib 2 mg daily	Upadacitinib 30 mg daily	0	--	6.8 (2.6, 11.1); high	6.8 (2.6, 11.1); high

© 2022 Drucker AM, et al. *JAMA Dermatology*.

Baricitinib 4 mg daily	Dupilumab	0	--	1.9 (0.5, 3.2); high	1.9 (0.5, 3.2); high
Baricitinib 4 mg daily	Placebo	4	-5.4 (-6.6, -4.2); high	--	-5.4 (-6.6, -4.2); high
Baricitinib 4 mg daily	Tralokinumab	0	--	-0.8 (-2.4, 0.7); high	-0.8 (-2.4, 0.7); high
Baricitinib 4 mg daily	Upadacitinib 15 mg daily	0	--	1.5 (-2.8, 5.8); high	1.5 (-2.8, 5.8); moderate[a]
Baricitinib 4 mg daily	Upadacitinib 30 mg daily	0	--	5.2 (1, 9.5); high	5.2 (1.0, 9.5); high
Dupilumab	Placebo	7	-7.3 (-8, -6.6); high	--	-7.3 (-8, -6.6); high
Dupilumab	Tralokinumab	0	--	-2.7 (-3.9, -1.5); high	-2.7 (-3.9, -1.5); high
Dupilumab	Upadacitinib 15 mg daily	0	--	-0.3 (-4.5, 3.9); high	-0.3 (-4.5, 3.9); moderate
Dupilumab	Upadacitinib 30 mg daily	0	--	3.3 (-0.8, 7.6); high	3.3 (-0.8, 7.6); moderate[a]
Tralokinumab	Upadacitinib 15 mg daily	0	--	2.4 (-1.8, 6.6); high	2.4 (-1.8, 6.6); moderate[a]
Tralokinumab	Upadacitinib 30 mg daily	0	--	6 (1.9, 10.3); high	6 (1.9, 10.3); high
Tralokinumab	Placebo	3	-4.6 (-5.6, -3.6); high	--	-4.6 (-5.6, -3.6); high
Upadacitinib 15 mg daily	Upadacitinib 30 mg daily	1	3.7 (-0.4, 7.7); high	--	3.7 (-0.4, 7.7); moderate[a]
Upadacitinib 15 mg daily	Placebo	1	-7 (-11.1, -2.9); high	--	-7 (-11.1, -2.9); high
Upadacitinib 30 mg daily	Placebo	1	-10.6 (-14.8, -6.6); high	--	-10.6 (-14.8, -6.6); high

Dose of dupilumab and tralokinumab is 600 mg then 300 mg every 2 weeks

The network meta-analysis certainty ratings start at the higher of the direct or indirect ratings and can then be rated down further for incoherence (based on the results of node splitting analyses) and imprecision.

a. Rated down for imprecision

© 2022 Drucker AM, et al. *JAMA Dermatology*.

eTable 6. League table with relative effect estimates for change in DLQI up to 16 weeks of treatment in adults for placebo and medications used in clinical practice or likely to be approved soon. Results for other pairwise comparisons in the network can be requested from the authors. Results are presented as change in DLQI (95% CrI). A positive effect estimate in a given cell favors the row-defining treatment. A negative effect estimate in a given cell favors the column-defining treatment. The bottom row contains the Surface Under the Cumulative Ranking (SUCRA) value for the column-defining treatment.

	Abrocitinib 100 mg daily	Abrocitinib 200 mg daily	Baricitinib 2 mg daily	Baricitinib 4 mg daily	Dupilumab 600 mg then 300 mg q2 weeks	Placebo	Tralokinumab 600 mg then 300 mg q2 weeks
Abrocitinib 100 mg daily		-2.1 (-2.9, -1.1)	1.1 (-0.1, 2.4)	-0.1 (-1.4, 1.2)	-1.5 (-2.5, -0.5)	3.4 (2.5, 4.3)	0.4 (-0.8, 1.8)
Abrocitinib 200 mg daily	2.1 (1.1, 2.9)		3.2 (2, 4.5)	1.9 (0.6, 3.3)	0.6 (-0.4, 1.5)	5.5 (4.6, 6.4)	2.5 (1.2, 3.8)
Baricitinib 2 mg daily	-1.1 (-2.4, 0.1)	-3.2 (-4.5, -2)		-1.3 (-2.2, -0.3)	-2.6 (-3.7, -1.6)	2.3 (1.4, 3.1)	-0.7 (-2, 0.6)
Baricitinib 4 mg daily	0.1 (-1.2, 1.4)	-1.9 (-3.3, -0.6)	1.3 (0.3, 2.2)		-1.4 (-2.5, -0.3)	3.5 (2.6, 4.4)	0.6 (-0.8, 1.9)
Dupilumab 600 mg then 300 mg q2 weeks	1.5 (0.5, 2.5)	-0.6 (-1.5, 0.4)	2.6 (1.6, 3.7)	1.4 (0.3, 2.5)		4.9 (4.3, 5.5)	2 (0.8, 3.1)
Placebo	-3.4 (-4.3, -2.5)	-5.5 (-6.4, -4.6)	-2.3 (-3.1, -1.4)	-3.5 (-4.4, -2.6)	-4.9 (-5.5, -4.3)		-3 (-3.9, -2)
Tralokinumab 600 mg then 300 mg q2 weeks	-0.4 (-1.8, 0.8)	-2.5 (-3.8, -1.2)	0.7 (-0.6, 2)	-0.6 (-1.9, 0.8)	-2 (-3.1, -0.8)	3 (2, 3.9)	
SUCRA	0.72	0.96	0.56	0.74	0.90	0.22	0.66

Dose of dupilumab and tralokinumab is 600 mg then 300 mg every 2 weeks

eTable 7. GRADE certainty ratings for change in DLQI up to 16 weeks of treatment in adults for placebo and medications used in clinical practice or likely to be approved soon. Results are presented as change in EASI (95% CrI). Negative effect estimates favor the treatment listed in the intervention column; positive effect estimates favor the comparator.

Intervention	Comparator	Head-to-head trials, n	Direct estimate (95% CrI); Certainty of Evidence	Indirect estimate (95% CrI); Certainty of Evidence	NMA Estimate (95% CrI); Certainty of Evidence
Abrocitinib 100 mg daily	Abrocitinib 200 mg daily	4	2.1 (1.1, 2.9); moderate*	--	2.1 (1.1, 2.9); moderate
Abrocitinib 100 mg daily	Baricitinib 2 mg daily	0	--	-1.1 (-2.4, 0.1); high	-1.1 (-2.4, 0.1); moderate[a]
Abrocitinib 100 mg daily	Baricitinib 4 mg daily	0	--	0.1 (-1.2, 1.4); high	0.1 (-1.2, 1.4); high
Abrocitinib 100 mg daily	Dupilumab	1	1.8 (0.5, 3.1); high	1.1 (-0.4, 2.5)	1.5 (0.5, 2.5); high
Abrocitinib 100 mg daily	Placebo	4	-3.4 (-4.3, -2.5); high	--	-3.4 (-4.3, -2.5); high
Abrocitinib 100 mg daily	Tralokinumab	0	--	-0.4 (-1.8, 0.8); high	-0.4 (-1.8, 0.8); high
Abrocitinib 200 mg daily	Baricitinib 2 mg daily	0	--	-3.2 (-4.5, -2); high	-3.2 (-4.5, -2); high
Abrocitinib 200 mg daily	Baricitinib 4 mg daily	0	--	-1.9 (-3.3, -0.6); high	-1.9 (-3.3, -0.6); high
Abrocitinib 200 mg daily	Dupilumab	1	-0.9 (-2.3, 0.5); high	-0.1 (-1.6, 1.4)	-0.6 (-1.5, 0.4); high
Abrocitinib 200 mg daily	Placebo	4	-5.5 (-6.4, -4.6); high	--	-5.5 (-6.4, -4.6); high
Abrocitinib 200 mg daily	Tralokinumab	0	--	-2.5 (-3.8, -1.2); high	-2.5 (-3.8, -1.2); high
Baricitinib 2 mg daily	Baricitinib 4 mg daily	5	1.3 (0.3, 2.2); moderate*	--	1.3 (0.3, 2.2); moderate
Baricitinib 2 mg daily	Dupilumab	0	--	2.6 (1.6, 3.7); high	2.6 (1.6, 3.7); high
Baricitinib 2 mg daily	Placebo	6	-2.3 (-3.1, -1.4); high	--	-2.3 (-3.1, -1.4); high
Baricitinib 2 mg daily	Tralokinumab	0	--	0.7 (-0.6, 2); high	0.7 (-0.6, 2); high
Baricitinib 4 mg daily	Dupilumab	0	--	1.4 (0.3, 2.5); high	1.4 (0.3, 2.5); high
Baricitinib 4 mg daily	Placebo	5	-3.5 (-4.4, -2.6); high	--	-3.5 (-4.4, -2.6); high
Baricitinib 4 mg daily	Tralokinumab	0	--	-0.6 (-1.9, 0.8); high	-0.6 (-1.9, 0.8); high
Dupilumab	Placebo	7	-4.9 (-5.5, -4.3); high	--	-4.9 (-5.5, -4.3); high
Dupilumab	Tralokinumab	0	--	-2 (-3.1, -0.8); high	-2 (-3.1, -0.8); high
Tralokinumab	Placebo	3	-3 (-3.9, -2); high	--	-3 (-3.9, -2); high

Dose of dupilumab and tralokinumab is 600 mg then 300 mg every 2 weeks
The network meta-analysis certainty ratings start at the higher of the direct or indirect ratings and can then be rated down further for incoherence (based on the results of node splitting analyses) and imprecision.
a. Rated down for imprecision
* Rated down for inconsistency

© 2022 Drucker AM, et al. *JAMA Dermatology*.

eTable 8. League table with relative effect estimates for change in peak pruritus numeric rating scales up to 16 weeks of treatment in adults for placebo and medications used in clinical practice or likely to be approved soon. Results for other pairwise comparisons in the network can be requested from the authors. Results are presented as change in the numeric rating scales (95% CrI). A positive effect estimate in a given cell favors the row-defining treatment. A negative effect estimate in a given cell favors the column-defining treatment. The bottom row contains the Surface Under the Cumulative Ranking (SUCRA) value for the column-defining treatment.

	Abrocitinib 100 mg daily	Abrocitinib 200 mg daily	Baricitinib 2 mg daily	Baricitinib 4 mg daily	Dupilumab 600 mg then 300 mg q2 weeks	Placebo	Tralokinumab 600 mg then 300 mg q2 weeks	Upadacitinib 15 mg once daily	Upadacitinib 30 mg once daily
Abrocitinib 100 mg daily		-0.8 (-1.3, -0.4)	0.3 (-0.4, 0.9)	-0.1 (-0.9, 0.6)	-0.4 (-1, 0.2)	1.6 (1, 2.1)	0.5 (-0.2, 1.1)	-0.8 (-1.5, -0.2)	-1.8 (-2.5, -1.1)
Abrocitinib 200 mg daily	0.8 (0.4, 1.3)		1.1 (0.4, 1.8)	0.7 (0, 1.4)	0.4 (-0.2, 1)	2.4 (1.9, 3)	1.3 (0.6, 1.9)	0 (-0.7, 0.7)	-1 (-1.7, -0.3)
Baricitinib 2 mg daily	-0.3 (-0.9, 0.4)	-1.1 (-1.8, -0.4)		-0.4 (-0.8, 0.1)	-0.7 (-1.2, -0.2)	1.3 (0.9, 1.7)	0.2 (-0.3, 0.7)	-1.1 (-1.7, -0.5)	-2.1 (-2.7, -1.5)
Baricitinib 4 mg daily	0.1 (-0.6, 0.9)	-0.7 (-1.4, 0)	0.4 (-0.1, 0.8)		-0.3 (-0.8, 0.2)	1.7 (1.3, 2.2)	0.6 (0.1, 1.1)	-0.7 (-1.3, -0.1)	-1.7 (-2.3, -1.1)
Dupilumab 600 mg then 300 mg q2 weeks	0.4 (-0.2, 1)	-0.4 (-1, 0.2)	0.7 (0.2, 1.2)	0.3 (-0.2, 0.8)		2 (1.8, 2.3)	0.9 (0.5, 1.3)	-0.4 (-0.9, 0.1)	-1.4 (-1.9, -0.9)
Placebo	-1.6 (-2.1, -1)	-2.4 (-3, -1.9)	-1.3 (-1.7, -0.9)	-1.7 (-2.2, -1.3)	-2 (-2.3, -1.8)		-1.1 (-1.4, -0.8)	-2.4 (-2.8, -2)	-3.4 (-3.8, -3)
Tralokinumab 600 mg then 300 mg q2 weeks	-0.5 (-1.1, 0.2)	-1.3 (-1.9, -0.6)	-0.2 (-0.7, 0.3)	-0.6 (-1.1, -0.1)	-0.9 (-1.3, -0.5)	1.1 (0.8, 1.4)		-1.3 (-1.8, -0.8)	-2.3 (-2.8, -1.8)
Upadacitinib 15 mg once daily	0.8 (0.2, 1.5)	0 (-0.7, 0.7)	1.1 (0.5, 1.7)	0.7 (0.1, 1.3)	0.4 (-0.1, 0.9)	2.4 (2, 2.8)	1.3 (0.8, 1.8)		-1 (-1.4, -0.6)
Upadacitinib 30 mg once daily	1.8 (1.1, 2.5)	1 (0.3, 1.7)	2.1 (1.5, 2.7)	1.7 (1.1, 2.3)	1.4 (0.9, 1.9)	3.4 (3, 3.8)	2.3 (1.8, 2.8)	1 (0.6, 1.4)	
SUCRA	0.47	0.74	0.39	0.50	0.60	0.09	0.33	0.74	0.92

Dose of dupilumab and tralokinumab is 600 mg then 300 mg every 2 weeks

a. Rated down for imprecision

© 2022 Drucker AM, et al. *JAMA Dermatology*.

eTable 9. GRADE certainty ratings for change in peak pruritus numeric rating scales up to 16 weeks of treatment in adults for placebo and medications used in clinical practice or likely to be approved soon. Results are presented as change in EASI (95% CrI). Negative effect estimates favor the treatment listed in the intervention column; positive effect estimates favor the comparator.

Intervention	Comparator	Head-to-head trials, n	Direct estimate (95% CrI); Certainty of Evidence	Indirect estimate (95% CrI); Certainty of Evidence	NMA Estimate (95% CrI); Certainty of Evidence
Abrocitinib 100 mg daily	Abrocitinib 200 mg daily	3	0.8 (0.4, 1.3); high	--	0.8 (0.4, 1.3); high
Abrocitinib 100 mg daily	Baricitinib 2 mg daily	0	--	-0.3 (-0.9, 0.4); high	-0.3 (-0.9, 0.4); high
Abrocitinib 100 mg daily	Baricitinib 4 mg daily	0	--	0.1 (-0.6, 0.9); high	0.1 (-0.6, 0.9); high
Abrocitinib 100 mg daily	Dupilumab	0	--	0.4 (-0.2, 1); high	0.4 (-0.2, 1); high
Abrocitinib 100 mg daily	Placebo	3	-1.6 (-2.1, -1); high	--	-1.6 (-2.1, -1); high
Abrocitinib 100 mg daily	Tralokinumab	0	--	-0.5 (-1.1, 0.2); high	-0.5 (-1.1, 0.2); high
Abrocitinib 100 mg daily	Upadacitinib 15 mg daily	0	--	0.8 (0.2, 1.5); high	0.8 (0.2, 1.5); high
Abrocitinib 100 mg daily	Upadacitinib 30 mg daily	0	--	1.8 (1.1, 2.5); high	1.8 (1.1, 2.5); high
Abrocitinib 200 mg daily	Baricitinib 2 mg daily	0	--	-1.1 (-1.8, -0.4); high	-1.1 (-1.8, -0.4); high
Abrocitinib 200 mg daily	Baricitinib 4 mg daily	0	--	-0.7 (-1.4, 0); high	-0.7 (-1.4, 0); high
Abrocitinib 200 mg daily	Dupilumab	0	--	-0.4 (-1, 0.2); high	-0.4 (-1, 0.2); high
Abrocitinib 200 mg daily	Placebo	3	-2.4 (-3, -1.9); high	--	-2.4 (-3, -1.9); high
Abrocitinib 200 mg daily	Tralokinumab	0	--	-1.3 (-1.9, -0.6); high	-1.3 (-1.9, -0.6); high
Abrocitinib 200 mg daily	Upadacitinib 15 mg daily	0	--	0 (-0.7, 0.7); high	0 (-0.7, 0.7); high
Abrocitinib 200 mg daily	Upadacitinib 30 mg daily	0	--	1 (0.3, 1.7); high	1 (0.3, 1.7); high
Baricitinib 2 mg daily	Baricitinib 4 mg daily	3	0.4 (-0.1, 0.8); high	--	0.4 (-0.1, 0.8); high
Baricitinib 2 mg daily	Dupilumab	0	--	0.7 (0.2, 1.2); high	0.7 (0.2, 1.2); high
Baricitinib 2 mg daily	Placebo	4	-1.3 (-1.7, -0.9); high	--	-1.3 (-1.7, -0.9); high
Baricitinib 2 mg daily	Tralokinumab	0	--	-0.2 (-0.7, 0.3); high	-0.2 (-0.7, 0.3); high
Baricitinib 2 mg daily	Upadacitinib 15 mg daily	0	--	1.1 (0.5, 1.7); high	1.1 (0.5, 1.7) high
Baricitinib 2 mg daily	Upadacitinib 30 mg daily	0	--	2.1 (1.5, 2.7); high	2.1 (1.5, 2.7); high
Baricitinib 4 mg daily	Dupilumab	0	--	0.3 (-0.2, 0.8); high	0.3 (-0.2, 0.8); high
Baricitinib 4 mg daily	Placebo	3	-1.7 (-2.2, -1.3); high	--	-1.7 (-2.2, -1.3); high
Baricitinib 4 mg daily	Tralokinumab	0	--	-0.6 (-1.1, -0.1); high	-0.6 (-1.1, -0.1); high
Baricitinib 4 mg daily	Upadacitinib 15 mg daily	0	--	0.7 (0.1, 1.3); high	0.7 (0.1, 1.3); high
Baricitinib 4 mg daily	Upadacitinib 30 mg daily	0	--	1.7 (1.1, 2.3); high	1.7 (1.1, 2.3); high

© 2022 Drucker AM, et al. *JAMA Dermatology*.

Dupilumab	Placebo	6	-2 (-2.3, -1.8); high	--	-2 (-2.3, -1.8); high
Dupilumab	Tralokinumab	0	--	-0.9 (-1.3, -0.5); high	-0.9 (-1.3, -0.5); high
Dupilumab	Upadacitinib 15 mg daily	0	--	0.4 (-0.1, 0.9); high	0.4 (-0.1, 0.9); high
Dupilumab	Upadacitinib 30 mg daily	0	--	1.4 (0.9, 1.9); high	1.4 (0.9, 1.9); high
Tralokinumab	Upadacitinib 15 mg daily	0	--	1.3 (0.8, 1.8); high	1.3 (0.8, 1.8); high
Tralokinumab	Upadacitinib 30 mg daily	0	--	2.3 (1.8, 2.8); high	2.3 (1.8, 2.8); high
Tralokinumab	Placebo	3	-1.1 (-1.4, -0.8); high	--	-1.1 (-1.4, -0.8); high
Upadacitinib 15 mg daily	Upadacitinib 30 mg daily	3	1 (0.6, 1.4); high	--	1 (0.6, 1.4); high
Upadacitinib 15 mg daily	Placebo	3	-2.4 (-2.8, -2); high	--	-2.4 (-2.8, -2); high
Upadacitinib 30 mg daily	Placebo	3	-3.4 (-3.8, -3); high	--	-3.4 (-3.8, -3); high

Dose of dupilumab and tralokinumab is 600 mg then 300 mg every 2 weeks

The network meta-analysis certainty ratings start at the higher of the direct or indirect ratings and can then be rated down further for incoherence (based on the results of node splitting analyses) and imprecision.

a. Rated down for imprecision

© 2022 Drucker AM, et al. *JAMA Dermatology*.

eFigure 1. Network graphs of studies included in the analysis of adults treated between 8 and 16 weeks for change in signs on the standardized mean difference scale. The width of each line connecting two treatments (nodes) is proportional to the number of head-to-head trials for that comparison.

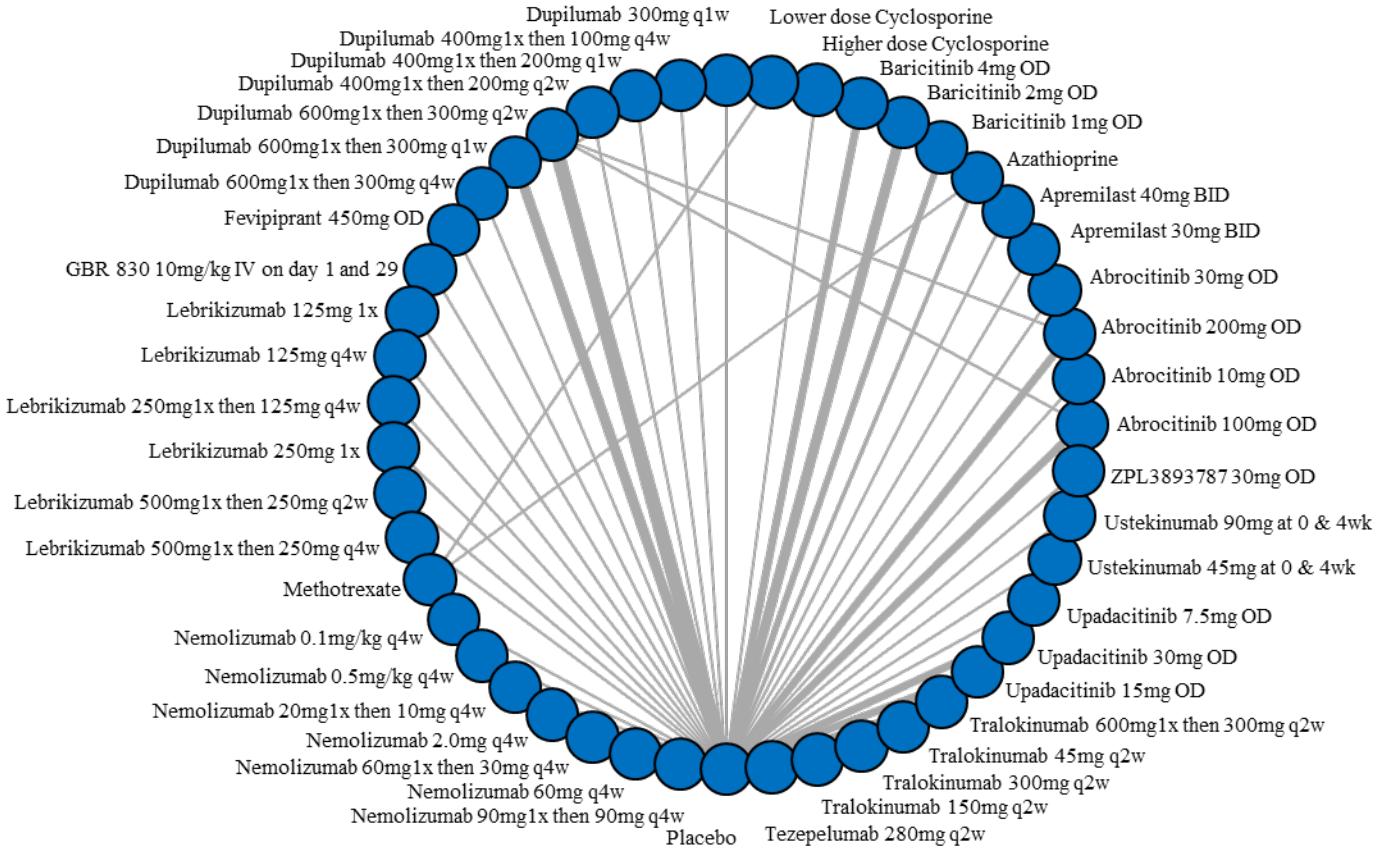

OD: once daily; q1w: once weekly; q2w: every 2 weeks; q4w: every 4 weeks.

© 2022 Drucker AM, et al. *JAMA Dermatology*.

eTable 10. League table with relative effect estimates for change in signs up to 16 weeks of treatment in adults for placebo and medications used in clinical practice or likely to be approved soon. Results for other pairwise comparisons in the network can be requested from the authors. Results are presented as change in standardized mean difference of signs scales (95% CrI). A positive effect estimate in a given cell favors the row-defining treatment. A negative effect estimate in a given cell favors the column-defining treatment. The bottom row contains the Surface Under the Cumulative Ranking (SUCRA) value for the column-defining treatment.

	Abrocitinib 100 mg daily	Abrocitinib 200 mg daily	Azathioprine	Baricitinib 2 mg daily	Baricitinib 4 mg daily	Cyclosporine (higher dose)	Cyclosporine (lower dose)	Dupilumab*	Methotrexate	Placebo	Tralokinumab*	Upadacitinib 15 mg daily	Upadacitinib 30 mg daily
Abrocitinib 100 mg daily		-0.4 (-0.6, -0.3)	0.1 (-0.3, 0.6)	0.4 (0.2, 0.6)	0.3 (0, 0.5)	-0.3 (-0.9, 0.4)	0 (-0.6, 0.7)	-0.1 (-0.3, 0)	0.1 (-0.5, 0.7)	0.8 (0.6, 0.9)	0.2 (0, 0.4)	-0.4 (-0.6, -0.2)	-0.6 (-0.8, -0.4)
Abrocitinib 200 mg daily	0.4 (0.3, 0.6)		0.6 (0.1, 1)	0.8 (0.6, 1)	0.7 (0.5, 0.9)	0.1 (-0.5, 0.8)	0.4 (-0.2, 1.1)	0.3 (0.1, 0.4)	0.5 (-0.1, 1.2)	1.2 (1, 1.3)	0.6 (0.4, 0.8)	0 (-0.2, 0.2)	-0.2 (-0.4, 0)
Azathioprine	-0.1 (-0.6, 0.3)	-0.6 (-1, -0.1)		0.2 (-0.2, 0.7)	0.1 (-0.3, 0.5)	-0.4 (-1, 0.2)	-0.1 (-0.7, 0.5)	-0.3 (-0.7, 0.1)	0 (-0.6, 0.5)	0.6 (0.2, 1)	0 (-0.4, 0.5)	-0.5 (-0.9, -0.1)	-0.8 (-1.2, -0.3)
Baricitinib 2 mg daily	-0.4 (-0.6, -0.2)	-0.8 (-1, -0.6)	-0.2 (-0.7, 0.2)		-0.1 (-0.3, 0)	-0.7 (-1.3, 0)	-0.4 (-1, 0.3)	-0.5 (-0.7, -0.3)	-0.3 (-0.9, 0.4)	0.4 (0.2, 0.5)	-0.2 (-0.4, 0)	-0.8 (-1, -0.6)	-1 (-1.2, -0.8)
Baricitinib 4 mg daily	-0.3 (-0.5, 0)	-0.7 (-0.9, -0.5)	-0.1 (-0.5, 0.3)	0.1 (0, 0.3)		-0.5 (-1.1, 0.1)	-0.2 (-0.9, 0.4)	-0.4 (-0.6, -0.2)	-0.1 (-0.8, 0.5)	0.5 (0.4, 0.7)	-0.1 (-0.3, 0.1)	-0.6 (-0.8, -0.4)	-0.9 (-1.1, -0.7)
Cyclosporine (higher dose)	0.3 (-0.4, 0.9)	-0.1 (-0.8, 0.5)	0.4 (-0.2, 1)	0.7 (0, 1.3)	0.5 (-0.1, 1.1)		0.3 (-0.1, 0.7)	0.2 (-0.5, 0.8)	0.4 (-0.2, 0.9)	1 (0.4, 1.6)	0.5 (-0.2, 1.1)	-0.1 (-0.7, 0.5)	-0.4 (-1, 0.3)
Cyclosporine (lower dose)	0 (-0.7, 0.6)	-0.4 (-1.1, 0.2)	0.1 (-0.5, 0.7)	0.4 (-0.3, 1)	0.2 (-0.4, 0.9)	-0.3 (-0.7, 0.1)		-0.2 (-0.8, 0.5)	0.1 (-0.4, 0.6)	0.7 (0.1, 1.4)	0.2 (-0.5, 0.8)	-0.4 (-1, 0.2)	-0.7 (-1.3, 0)
Dupilumab*	0.1 (0, 0.3)	-0.3 (-0.4, -0.1)	0.3 (-0.1, 0.7)	0.5 (0.3, 0.7)	0.4 (0.2, 0.6)	-0.2 (-0.8, 0.5)	0.2 (-0.5, 0.8)		0.2 (-0.4, 0.9)	0.9 (0.8, 1)	0.3 (0.1, 0.5)	-0.2 (-0.4, -0.1)	-0.5 (-0.7, -0.3)
Methotrexate	-0.1 (-0.7, 0.5)	-0.5 (-1.2, 0.1)	0 (-0.5, 0.6)	0.3 (-0.4, 0.9)	0.1 (-0.5, 0.8)	-0.4 (-0.9, 0.2)	-0.1 (-0.6, 0.4)	-0.2 (-0.9, 0.4)		0.6 (0, 1.3)	0.1 (-0.6, 0.7)	-0.5 (-1.1, 0.1)	-0.8 (-1.4, -0.1)
Placebo	-0.8 (-0.9, -0.6)	-1.2 (-1.3, -1)	-0.6 (-1, -0.2)	-0.4 (-0.5, -0.2)	-0.5 (-0.7, -0.4)	-1 (-1.6, -0.4)	-0.7 (-1.4, -0.1)	-0.9 (-1, -0.8)	-0.6 (-1.3, 0)		-0.6 (-0.7, -0.4)	-1.1 (-1.3, -1)	-1.4 (-1.5, -1.3)
Tralokinumab*	-0.2 (-0.4, 0)	-0.6 (-0.8, -0.4)	0 (-0.5, 0.4)	0.2 (0, 0.4)	0.1 (-0.1, 0.3)	-0.5 (-1.1, 0.2)	-0.2 (-0.8, 0.5)	-0.3 (-0.5, -0.1)	-0.1 (-0.7, 0.6)	0.6 (0.4, 0.7)		-0.6 (-0.7, -0.4)	-0.8 (-1, -0.6)
Upadacitinib 15 mg daily	0.4 (0.2, 0.6)	0 (-0.2, 0.2)	0.5 (0.1, 0.9)	0.8 (0.6, 1)	0.6 (0.4, 0.8)	0.1 (-0.5, 0.7)	0.4 (-0.2, 1)	0.2 (0.1, 0.4)	0.5 (-0.1, 1.1)	1.1 (1, 1.3)	0.6 (0.4, 0.7)		-0.3 (-0.4, -0.1)
Upadacitinib 30 mg daily	0.6 (0.4, 0.8)	0.2 (0, 0.4)	0.8 (0.3, 1.2)	1 (0.8, 1.2)	0.9 (0.7, 1.1)	0.4 (-0.3, 1)	0.7 (0, 1.3)	0.5 (0.3, 0.7)	0.8 (0.1, 1.4)	1.4 (1.3, 1.5)	0.8 (0.6, 1)	0.3 (0.1, 0.4)	
SUCRA	0.72	0.91	0.59	0.38	0.51	0.83	0.66	0.80	0.60	0.09	0.58	0.90	0.96

*Dose of dupilumab and tralokinumab is 600 mg then 300 mg every 2 weeks

© 2022 Drucker AM, et al. *JAMA Dermatology*.

eTable 11. GRADE certainty ratings for change in the standardized mean difference of clinical signs up to 16 weeks of treatment in adults for placebo and medications used in clinical practice or likely to be approved soon. Results are presented standardized mean difference (95% CrI). Negative effect estimates favor the treatment listed in the intervention column; positive effect estimates favor the comparator.

Intervention	Comparator	Head-to-head trials, n	Direct estimate (95% CrI); Certainty of Evidence	Indirect estimate (95% CrI); Certainty of Evidence	NMA Estimate (95% CrI); Certainty of Evidence
Abrocitinib 100 mg daily	Abrocitinib 200 mg daily	4	0.4 (0.3, 0.6); high	--	0.4 (0.3, 0.6); high
Abrocitinib 100 mg daily	Azathioprine	0	--	-0.1 (-0.6, 0.3); moderate[a]	-0.1 (-0.6, 0.3); low[b]
Abrocitinib 100 mg daily	Baricitinib 2 mg daily	0	--	-0.4 (-0.6, -0.2); high	-0.4 (-0.6, -0.2); high
Abrocitinib 100 mg daily	Baricitinib 4 mg daily	0	--	-0.3 (-0.5, 0); high	-0.3 (-0.5, 0); high
Abrocitinib 100 mg daily	Cyclosporine (higher dose)	0	--	0.3 (-0.4, 0.9); moderate[a]	0.3 (-0.4, 0.9); low[b]
Abrocitinib 100 mg daily	Cyclosporine (lower dose)	0	--	0 (-0.7, 0.6); moderate[a]	0 (-0.7, 0.6); low[b]
Abrocitinib 100 mg daily	Dupilumab	1	0.1 (-0.1, 0.3); high	0.2 (0.0, 0.4); high	0.1 (0, 0.3); high
Abrocitinib 100 mg daily	Methotrexate	0	--	-0.1 (-0.7, 0.5); moderate[a]	-0.1 (-0.7, 0.5); low[b]
Abrocitinib 100 mg daily	Placebo	4	-0.8 (-0.9, -0.6); high	--	-0.8 (-0.9, -0.6); high
Abrocitinib 100 mg daily	Tralokinumab	0	--	-0.2 (-0.4, 0); high	-0.2 (-0.4, 0); high
Abrocitinib 100 mg daily	Upadacitinib 15 mg daily	0	--	0.4 (0.2, 0.6); high	0.4 (0.2, 0.6); high
Abrocitinib 100 mg daily	Upadacitinib 30 mg daily	0	--	0.6 (0.4, 0.8); high	0.6 (0.4, 0.8); high
Abrocitinib 200 mg daily	Azathioprine	0	--	-0.6 (-1, -0.1); high	-0.6 (-1, -0.1); high
Abrocitinib 200 mg daily	Baricitinib 2 mg daily	0	--	-0.8 (-1, -0.6); high	-0.8 (-1, -0.6); high
Abrocitinib 200 mg daily	Baricitinib 4 mg daily	0	--	-0.7 (-0.9, -0.5); high	-0.7 (-0.9, -0.5); high
Abrocitinib 200 mg daily	Cyclosporine (higher dose)	0	--	-0.1 (-0.8, 0.5); moderate[a]	-0.1 (-0.8, 0.5); low[b]
Abrocitinib 200 mg daily	Cyclosporine (lower dose)	0	--	-0.4 (-1.1, 0.2); moderate[a]	-0.4 (-1.1, 0.2); low[b]
Abrocitinib 200 mg daily	Dupilumab	1	-0.3 (-0.5, -0.1); high	-0.2 (-0.5, 0); high	-0.3 (-0.4, -0.1); high
Abrocitinib 200 mg daily	Methotrexate	0	--	-0.5 (-1.2, 0.1); moderate[a]	-0.5 (-1.2, 0.1); low[b]

© 2022 Drucker AM, et al. *JAMA Dermatology*.

Abrocitinib 200 mg daily	Placebo	4	-1.2 (-1.3, -1); high	--	-1.2 (-1.3, -1); high
Abrocitinib 200 mg daily	Tralokinumab	0	--	-0.6 (-0.8, -0.4); high	-0.6 (-0.8, -0.4); high
Abrocitinib 200 mg daily	Upadacitinib 15 mg daily	0	--	0 (-0.2, 0.2); high	0 (-0.2, 0.2); high
Abrocitinib 200 mg daily	Upadacitinib 30 mg daily	0	--	0.2 (0, 0.4); high	0.2 (0, 0.4); moderate[b]
Azathioprine	Baricitinib 2 mg daily	0	--	-0.2 (-0.7, 0.2); moderate[a]	-0.2 (-0.7, 0.2); low[b]
Azathioprine	Baricitinib 4 mg daily	0	--	-0.1 (-0.5, 0.3); moderate[a]	-0.1 (-0.5, 0.3); moderate
Azathioprine	Cyclosporine (higher dose)	0	--	0.4 (-0.2, 1); moderate[a]	0.4 (-0.2, 1); low[b]
Azathioprine	Cyclosporine (lower dose)	0	--	0.1 (-0.5, 0.7); moderate[a]	0.1 (-0.5, 0.7); moderate
Azathioprine	Dupilumab	0	--	0.3 (-0.1, 0.7); moderate[a]	0.3 (-0.1, 0.7); low[b]
Azathioprine	Methotrexate	1	0 (-0.6, 0.6); moderate[a]	0.1 (-1.0, 1.2); moderate[a]	0 (-0.5, 0.6); moderate
Azathioprine	Placebo	1	-0.6 (-1.1, -0.2); moderate[a]	-0.7 (-1.8, 0.5); moderate[a]	-0.6 (-1.0, -0.2); moderate
Azathioprine	Tralokinumab	0	--	0 (-0.5, 0.4); moderate[a]	0 (-0.5, 0.4); moderate
Azathioprine	Upadacitinib 15 mg daily	0	--	0.5 (0.1, 0.9); moderate[a]	0.5 (0.1, 0.9); moderate
Azathioprine	Upadacitinib 30 mg daily	0	--	0.8 (0.3, 1.2); moderate[a]	0.8 (0.3, 1.2); moderate
Baricitinib 2 mg daily	Baricitinib 4 mg daily	4	0.1 (0, 0.3); moderate*	--	0.1 (0, 0.3); moderate
Baricitinib 2 mg daily	Cyclosporine (higher dose)	0	--	0.7 (0, 1.3); moderate[a]	0.7 (0, 1.3); low[b]
Baricitinib 2 mg daily	Cyclosporine (lower dose)	0	--	0.4 (-0.3, 1); moderate[a]	0.4 (-0.3, 1); low[b]
Baricitinib 2 mg daily	Dupilumab	0	--	0.5 (0.3, 0.7); high	0.5 (0.3, 0.7); high
Baricitinib 2 mg daily	Methotrexate	0	--	0.3 (-0.4, 0.9); moderate[a]	0.3 (-0.4, 0.9); low[b]
Baricitinib 2 mg daily	Placebo	5	-0.4 (-0.5, -0.2); high	--	-0.4 (-0.5, -0.2); high
Baricitinib 2 mg daily	Tralokinumab	0	--	0.2 (0, 0.4); high	0.2 (0, 0.4); moderate[b]

© 2022 Drucker AM, et al. *JAMA Dermatology*.

Baricitinib 2 mg daily	Upadacitinib 15 mg daily	0	--	0.8 (0.6, 1); high	0.8 (0.6, 1); high
Baricitinib 2 mg daily	Upadacitinib 30 mg daily	0	--	1 (0.8, 1.2); high	1 (0.8, 1.2); high
Baricitinib 4 mg daily	Cyclosporine (higher dose)	0	--	0.5 (-0.1, 1.1); moderate[a]	0.5 (-0.1, 1.1); low[b]
Baricitinib 4 mg daily	Cyclosporine (lower dose)	0	--	0.2 (-0.4, 0.9); moderate[a]	0.2 (-0.4, 0.9); low[b]
Baricitinib 4 mg daily	Dupilumab	0	--	0.4 (0.2, 0.6); high	0.4 (0.2, 0.6); high
Baricitinib 4 mg daily	Methotrexate	0	--	0.1 (-0.5, 0.8); moderate[a]	0.1 (-0.5, 0.8); moderate
Baricitinib 4 mg daily	Placebo	4	-0.5 (-0.7, -0.4); high	--	-0.5 (-0.7, -0.4); high
Baricitinib 4 mg daily	Tralokinumab	0	--	0.1 (-0.1, 0.3); high	0.1 (-0.1, 0.3); high
Baricitinib 4 mg daily	Upadacitinib 15 mg daily	0	--	0.6 (0.4, 0.8); high	0.6 (0.4, 0.8); high
Baricitinib 4 mg daily	Upadacitinib 30 mg daily	0	--	0.9 (0.7, 1.1); high	0.9 (0.7, 1.1); high
Cyclosporine (higher dose)	Cyclosporine (lower dose)	1	-0.3 (-0.7, 0.1); moderate[a]	-0.4 (-1.5, 0.8); moderate[a]	-0.3 (-0.7, 0.1); low[b]
Cyclosporine (higher dose)	Dupilumab	0	--	-0.2 (-0.8, 0.5); high	-0.2 (-0.8, 0.5); low[b]
Cyclosporine (higher dose)	Methotrexate	0	--	-0.4 (-0.9, 0.2); moderate[a]	-0.4 (-0.9, 0.2); low[b]
Cyclosporine (higher dose)	Placebo	1	-1 (-1.8, -0.3); moderate[a]	-1 (-2.1, 0); moderate[a]	-1 (-1.6, -0.4; moderate
Cyclosporine (higher dose)	Tralokinumab	0	--	-0.5 (-1.1, 0.2); moderate[a]	-0.5 (-1.1, 0.2); low[b]
Cyclosporine (higher dose)	Upadacitinib 15 mg daily	0	--	0.1 (-0.5, 0.7); moderate[a]	0.1 (-0.5, 0.7); moderate
Cyclosporine (higher dose)	Upadacitinib 30 mg daily	0	--	0.4 (-0.3, 1); moderate[a]	0.4 (-0.3, 1); low[b]
Cyclosporine (lower dose)	Dupilumab	0	--	0.2 (-0.5, 0.8); moderate[a]	0.2 (-0.5, 0.8); low[b]
Cyclosporine (lower dose)	Methotrexate	1	-0.1 (-0.6, 0.5); moderate[a]	-0.1 (-1.2, 1.0); moderate[a]	-0.1 (-0.6, 0.4); moderate
Cyclosporine (lower dose)	Placebo	0	--	-0.7 (-1.4, -0.1); moderate[a]	-0.7 (-1.4, -0.1); moderate
Cyclosporine (lower dose)	Tralokinumab	0	--	-0.2 (-0.8, 0.5); moderate[a]	-0.2 (-0.8, 0.5); low[b]
Cyclosporine (lower dose)	Upadacitinib 15 mg daily	0	--	0.4 (-0.2, 1); moderate[a]	0.4 (-0.2, 1); low[b]

© 2022 Drucker AM, et al. *JAMA Dermatology*.

Cyclosporine (lower dose)	Upadacitinib 30 mg daily	0	--	0.7 (0, 1.3); moderate[a]	0.7 (0, 1.3); low[b]
Dupilumab	Methotrexate	0	--	-0.2 (-0.9, 0.4); moderate[a]	-0.2 (-0.9, 0.4); low[b]
Dupilumab	Placebo	7	-0.9 (-1, -0.8); high	--	-0.9 (-1, -0.8); high
Dupilumab	Tralokinumab	0	--	-0.3 (-0.5, -0.1); high	-0.3 (-0.5, -0.1); high
Dupilumab	Upadacitinib 15 mg daily	0	--	0.2 (0.1, 0.4); high	0.2 (0.1, 0.4); high
Dupilumab	Upadacitinib 30 mg daily	0	--	0.5 (0.3, 0.7); high	0.5 (0.3, 0.7); high
Methotrexate	Placebo	0	--	-0.6 (-1.3, 0); moderate[a]	-0.6 (-1.3, 0); low[b]
Methotrexate	Tralokinumab	0	--	-0.1 (-0.7, 0.6); moderate[a]	-0.1 (-0.7, 0.6); moderate
Methotrexate	Upadacitinib 15 mg daily	0	--	0.5 (-0.1, 1.1); moderate[a]	0.5 (-0.1, 1.1); low[b]
Methotrexate	Upadacitinib 30 mg daily	0	--	0.8 (0.1, 1.4); moderate[a]	0.8 (0.1, 1.4); moderate
Tralokinumab	Placebo	3	-0.6 (-0.7, -0.4); high	--	-0.6 (-0.7, -0.4); high
Tralokinumab	Upadacitinib 15 mg daily	0	--	0.6 (0.4, 0.7); high	0.6 (0.4, 0.7); high
Tralokinumab	Upadacitinib 30 mg daily	0	--	0.8 (0.6, 1); high	0.8 (0.6, 1); high
Upadacitinib 15 mg daily	Upadacitinib 30 mg daily	4	0.3 (0.1, 0.4); high	--	0.3 (0.1, 0.4); high
Upadacitinib 15 mg daily	Placebo	4	-1.1 (-1.3, -1); high	--	-1.1 (-1.3, -1); high
Upadacitinib 30 mg daily	Placebo	4	-1.4 (-1.5, -1.3); high	--	-1.4 (-1.5, -1.3); high

Dose of dupilumab and tralokinumab is 600 mg then 300 mg every 2 weeks

The network meta-analysis certainty ratings start at the higher of the direct or indirect ratings and can then be rated down further for incoherence (based on the results of node splitting analyses) and imprecision.

* Rated down for inconsistency
a. Rated down for significant issues related to risk of bias in included trials
b. Rated down for imprecision

© 2022 Drucker AM, et al. *JAMA Dermatology*.

eFigure 2. Network graphs of studies included in the analysis of adults treated between 8 and 16 weeks for change in quality of life on the standardized mean difference scale. The width of each line connecting two treatments (nodes) is proportional to the number of head-to-head trials for that comparison.

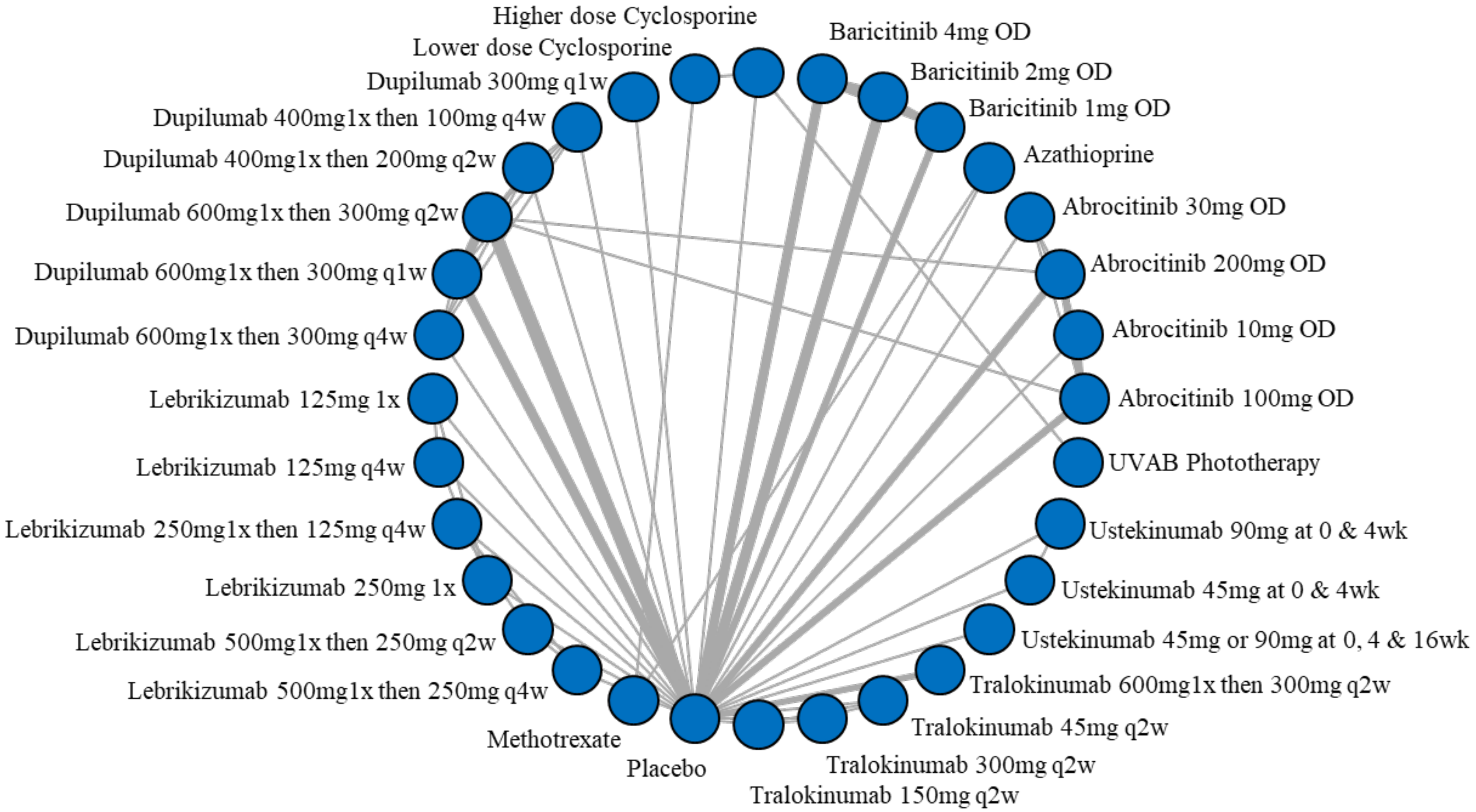

OD: once daily; q1w: once weekly; q2w: every 2 weeks; q4w: every 4 weeks.

© 2022 Drucker AM, et al. *JAMA Dermatology*.

eTable 12. League table with relative effect estimates for change in quality of life up to 16 weeks of treatment in adults for placebo and medications used in clinical practice or likely to be approved soon. Results for other pairwise comparisons in the network can be requested from the authors. Results are presented as change in standardized mean difference of quality of life scales (95% CrI). A positive effect estimate in a given cell favors the row-defining treatment. A negative effect estimate in a given cell favors the column-defining treatment. The bottom row contains the Surface Under the Cumulative Ranking (SUCRA) value for the column-defining treatment.

	Abrocitinib 100 mg daily	Abrocitinib 200 mg daily	Azathioprine	Baricitinib 2 mg daily	Baricitinib 4 mg daily	Cyclosporine (higher dose)	Cyclosporine (lower dose)	Dupilumab*	Methotrexate	Placebo	Tralokinumab*
Abrocitinib 100 mg daily		-0.3 (-0.5, -0.2)	0.2 (-0.4, 0.7)	0.2 (0, 0.4)	0 (-0.3, 0.2)	-0.1 (-0.8, 0.5)	0.1 (-0.6, 0.8)	-0.3 (-0.4, -0.1)	0.2 (-0.5, 0.8)	0.6 (0.4, 0.7)	0.2 (-0.1, 0.4)
Abrocitinib 200 mg daily	0.3 (0.2, 0.5)		0.5 (0, 1.1)	0.5 (0.3, 0.8)	0.3 (0, 0.5)	0.2 (-0.4, 0.8)	0.4 (-0.3, 1.1)	0.1 (-0.1, 0.3)	0.5 (-0.2, 1.2)	0.9 (0.7, 1.1)	0.5 (0.3, 0.7)
Azathioprine	-0.2 (-0.7, 0.4)	-0.5 (-1.1, 0)		0 (-0.5, 0.6)	-0.2 (-0.8, 0.3)	-0.3 (-1, 0.3)	-0.1 (-0.7, 0.6)	-0.4 (-1, 0.1)	0 (-0.6, 0.5)	0.4 (-0.1, 0.9)	0 (-0.6, 0.5)
Baricitinib 2 mg daily	-0.2 (-0.4, 0)	-0.5 (-0.8, -0.3)	0 (-0.6, 0.5)		-0.3 (-0.4, -0.1)	-0.3 (-1, 0.3)	-0.1 (-0.8, 0.5)	-0.5 (-0.7, -0.3)	0 (-0.7, 0.6)	0.4 (0.2, 0.5)	0 (-0.3, 0.2)
Baricitinib 4 mg daily	0 (-0.2, 0.3)	-0.3 (-0.5, 0)	0.2 (-0.3, 0.8)	0.3 (0.1, 0.4)		-0.1 (-0.7, 0.5)	0.1 (-0.5, 0.8)	-0.2 (-0.4, 0)	0.2 (-0.5, 0.9)	0.6 (0.4, 0.8)	0.2 (0, 0.4)
Cyclosporine (higher dose)	0.1 (-0.5, 0.8)	-0.2 (-0.8, 0.4)	0.3 (-0.3, 1)	0.3 (-0.3, 1)	0.1 (-0.5, 0.7)		0.2 (-0.2, 0.6)	-0.1 (-0.7, 0.5)	0.3 (-0.3, 0.9)	0.7 (0.1, 1.3)	0.3 (-0.3, 0.9)
Cyclosporine (lower dose)	-0.1 (-0.8, 0.6)	-0.4 (-1.1, 0.3)	0.1 (-0.6, 0.7)	0.1 (-0.5, 0.8)	-0.1 (-0.8, 0.5)	-0.2 (-0.6, 0.2)		-0.3 (-1, 0.3)	0.1 (-0.4, 0.6)	0.5 (-0.2, 1.1)	0.1 (-0.6, 0.8)
Dupilumab*	0.3 (0.1, 0.4)	-0.1 (-0.3, 0.1)	0.4 (-0.1, 1)	0.5 (0.3, 0.7)	0.2 (0, 0.4)	0.1 (-0.5, 0.7)	0.3 (-0.3, 1)		0.4 (-0.3, 1.1)	0.8 (0.7, 0.9)	0.4 (0.2, 0.6)
Methotrexate	-0.2 (-0.8, 0.5)	-0.5 (-1.2, 0.2)	0 (-0.5, 0.6)	0 (-0.6, 0.7)	-0.2 (-0.9, 0.5)	-0.3 (-0.9, 0.3)	-0.1 (-0.6, 0.4)	-0.4 (-1.1, 0.3)		0.4 (-0.3, 1.1)	0 (-0.7, 0.7)
Placebo	-0.6 (-0.7, -0.4)	-0.9 (-1.1, -0.7)	-0.4 (-0.9, 0.1)	-0.4 (-0.5, -0.2)	-0.6 (-0.8, -0.4)	-0.7 (-1.3, -0.1)	-0.5 (-1.1, 0.2)	-0.8 (-0.9, -0.7)	-0.4 (-1.1, 0.3)		-0.4 (-0.6, -0.3)
Tralokinumab*	-0.2 (-0.4, 0.1)	-0.5 (-0.7, -0.3)	0 (-0.5, 0.6)	0 (-0.2, 0.3)	-0.2 (-0.4, 0)	-0.3 (-0.9, 0.3)	-0.1 (-0.8, 0.6)	-0.4 (-0.6, -0.2)	0 (-0.7, 0.7)	0.4 (0.3, 0.6)	
SUCRA	0.70	0.93	0.53	0.51	0.74	0.78	0.60	0.88	0.53	0.18	0.56

*Dose of dupilumab and tralokinumab is 600 mg then 300 mg every 2 weeks

© 2022 Drucker AM, et al. *JAMA Dermatology*.

eTable 13. GRADE certainty ratings for change in the standardized mean difference of quality of life up to 16 weeks of treatment in adults for placebo and medications used in clinical practice or likely to be approved soon. Results are presented standardized mean difference (95% CrI). Negative effect estimates favor the treatment listed in the intervention column; positive effect estimates favor the comparator.

Intervention	Comparator	Head-to-head trials, n	Direct estimate (95% CrI); Certainty of Evidence	Indirect estimate (95% CrI); Certainty of Evidence	NMA Estimate (95% CrI); Certainty of Evidence
Abrocitinib 100 mg daily	Abrocitinib 200 mg daily	4	0.3 (0.2, 0.5); moderate*	--	0.3 (0.2, 0.5); moderate
Abrocitinib 100 mg daily	Azathioprine	0	--	-0.2 (-0.7, 0.4); moderate[a]	-0.2 (-0.7, 0.4); low[b]
Abrocitinib 100 mg daily	Baricitinib 2 mg daily	0	--	-0.2 (-0.4, 0); high	-0.2 (-0.4, 0); moderate[b]
Abrocitinib 100 mg daily	Baricitinib 4 mg daily	0	--	0 (-0.2, 0.3); high	0 (-0.2, 0.3); high
Abrocitinib 100 mg daily	Cyclosporine (higher dose)	0	--	0.1 (-0.5, 0.8); moderate[a]	0.1 (-0.5, 0.8); low[b]
Abrocitinib 100 mg daily	Cyclosporine (lower dose)	0	--	-0.1 (-0.8, 0.6); moderate[a]	-0.1 (-0.8, 0.6); low[b]
Abrocitinib 100 mg daily	Dupilumab	1	0.4 (0.1, 0.6); high	0.2 (-0.1, 0.4); high	0.3 (0.1, 0.4); high
Abrocitinib 100 mg daily	Methotrexate	0	--	0.2 (-0.8, 0.5); moderate[a]	-0.2 (-0.8, 0.5); low[b]
Abrocitinib 100 mg daily	Placebo	4	-0.6 (-0.7, -0.4); high	--	-0.6 (-0.7, -0.4); high
Abrocitinib 100 mg daily	Tralokinumab	0	--	-0.2 (-0.4, 0.1); high	-0.2 (-0.4, 0.1); moderate[b]
Abrocitinib 200 mg daily	Azathioprine	0	--	-0.5 (-1.1, 0); moderate[a]	-0.5 (-1.1, 0); low[b]
Abrocitinib 200 mg daily	Baricitinib 2 mg daily	0	--	-0.5 (-0.8, -0.3); high	-0.5 (-0.8, -0.3); high
Abrocitinib 200 mg daily	Baricitinib 4 mg daily	0	--	-0.3 (-0.5, 0); high	-0.3 (-0.5, 0); moderate[b]
Abrocitinib 200 mg daily	Cyclosporine (higher dose)	0	--	-0.2 (-0.8, 0.4); moderate[a]	-0.2 (-0.8, 0.4); low[b]
Abrocitinib 200 mg daily	Cyclosporine (lower dose)	0	--	-0.4 (-1.1, 0.3); moderate[a]	-0.4 (-1.1, 0.3); low[b]

© 2022 Drucker AM, et al. *JAMA Dermatology*.

Abrocitinib 200 mg daily	Dupilumab	1	-0.2 (-0.4, 0); high	0 (-3, 3); high	-0.1 (-0.3, 0.1); high
Abrocitinib 200 mg daily	Methotrexate	0	--	-0.5 (-1.2, 0.2); moderate[a]	-0.5 (-1.2, 0.2); low[b]
Abrocitinib 200 mg daily	Placebo	4	-0.9 (-1.1, -0.7); high	--	-0.9 (-1.1, -0.7); high
Abrocitinib 200 mg daily	Tralokinumab	0	--	-0.5 (-0.7, -0.3); high	-0.5 (-0.7, -0.3); high
Azathioprine	Baricitinib 2 mg daily	0	--	0 (-0.6, 0.5); moderate[a]	0 (-0.6, 0.5); moderate
Azathioprine	Baricitinib 4 mg daily	0	--	0.2 (-0.3, 0.8); moderate[a]	0.2 (-0.3, 0.8); low[b]
Azathioprine	Cyclosporine (higher dose)	0	--	0.3 (-0.3, 1); moderate[a]	0.3 (-0.3, 1); low[b]
Azathioprine	Cyclosporine (lower dose)	0	--	0.1 (-0.6, 0.7); moderate[a]	0.1 (-0.6, 0.7); moderate
Azathioprine	Dupilumab	0	--	0.4 (-0.1, 1); moderate[a]	0.4 (-0.1, 1); low[b]
Azathioprine	Methotrexate	1	0.2 (-0.4, 0.9); moderate[a]	-0.7 (-1.9, 0.4); moderate[a]	0 (-0.5, 0.6); moderate
Azathioprine	Placebo	1	-0.6 (-1.2, 0); moderate[a]	0.4 (-0.8, 1.6); moderate[a]	-0.4 (-0.9, 0.1); low[b]
Azathioprine	Tralokinumab	0	--	0 (-0.5, 0.6); moderate[a]	0 (-0.5, 0.6); moderate
Baricitinib 2 mg daily	Baricitinib 4 mg daily	5	0.3 (0.1, 0.4); high	--	0.3 (0.1, 0.4); high
Baricitinib 2 mg daily	Cyclosporine (higher dose)	0	--	0.3 (-0.3, 1); moderate[a]	0.3 (-0.3, 1); low[b]
Baricitinib 2 mg daily	Cyclosporine (lower dose)	0	--	0.1 (-0.5, 0.8); moderate[a]	0.1 (-0.5, 0.8); low[b]
Baricitinib 2 mg daily	Dupilumab	0	--	0.5 (0.3, 0.7); high	0.5 (0.3, 0.7); high
Baricitinib 2 mg daily	Methotrexate	0	--	0 (-0.6, 0.7); moderate[a]	0 (-0.6, 0.7); moderate
Baricitinib 2 mg daily	Placebo	6	-0.4 (-0.5, -0.2); high	--	-0.4 (-0.5, -0.2); high
Baricitinib 2 mg daily	Tralokinumab	0	--	0 (-0.2, 0.3); high	0 (-0.2, 0.3); high
Baricitinib 4 mg daily	Cyclosporine (higher dose)	0	--	0.1 (-0.5, 0.7); moderate[a]	0.1 (-0.5, 0.7); moderate
Baricitinib 4 mg daily	Cyclosporine (lower dose)	0	--	-0.1 (-0.8, 0.5); moderate[a]	-0.1 (-0.8, 0.5); low[b]

© 2022 Drucker AM, et al. *JAMA Dermatology*.

Baricitinib 4 mg daily	Dupilumab	0	--	0.2 (0, 0.4); high	0.2 (0, 0.4); moderate[b]
Baricitinib 4 mg daily	Methotrexate	0	--	-0.2 (-0.9, 0.5); moderate[a]	-0.2 (-0.9, 0.5); low[b]
Baricitinib 4 mg daily	Placebo	5	-0.6 (-0.8, -0.4); high	--	-0.6 (-0.8, -0.4); high
Baricitinib 4 mg daily	Tralokinumab	0	--	-0.6 (-0.8, -0.4); high	-0.2 (-0.4, 0); moderate[b]
Cyclosporine (higher dose)	Cyclosporine (lower dose)	1	-0.3 (-0.8, 0.1); moderate[a]	0.6 (-0.6, 1.9); moderate[a]	-0.2 (-0.6, 0.2); low[b]
Cyclosporine (higher dose)	Dupilumab	0	--	0.1 (-0.5, 0.7); moderate[a]	0.1 (-0.5, 0.7); moderate
Cyclosporine (higher dose)	Methotrexate	0	--	-0.3 (-0.9, 0.3); moderate[a]	-0.3 (-0.9, 0.3); low[b]
Cyclosporine (higher dose)	Placebo	1	-0.4 (-1.1, 0.3); moderate[a]	-1.4 (-2.5, 0.3); moderate[a]	-0.7 (-1.3, -0.1); moderate
Cyclosporine (higher dose)	Tralokinumab	0	--	-0.3 (-0.9, 0.3); moderate[a]	-0.3 (-0.9, 0.3); low[b]
Cyclosporine (lower dose)	Dupilumab	0	--	0.3 (-0.3, 1); moderate[a]	0.3 (-0.3, 1); low[b]
Cyclosporine (lower dose)	Methotrexate	1	-0.2 (-0.8, 0.3); moderate[a]	0.7 (-0.5, 1.9); moderate[a]	-0.1 (-0.6, 0.4); moderate
Cyclosporine (lower dose)	Placebo	0	--	-0.5 (-1.1, 0.2); moderate[a]	-0.5 (-1.1, 0.2); low[b]
Cyclosporine (lower dose)	Tralokinumab	0	--	-0.1 (-0.8, 0.6); moderate[a]	-0.1 (-0.8, 0.6); low[b]
Dupilumab	Methotrexate	0	--	-0.4 (-1.1, 0.3); moderate[a]	-0.4 (-1.1, 0.3); low[b]
Dupilumab	Placebo	7	-0.8 (-0.9, -0.7); high	--	-0.8 (-0.9, -0.7); high
Dupilumab	Tralokinumab	0	--	-0.4 (-0.6, -0.2); high	-0.4 (-0.6, -0.2); high
Methotrexate	Placebo	0	--	-0.4 (-1.1, 0.3); moderate[a]	-0.4 (-1.1, 0.3); low[b]
Methotrexate	Tralokinumab	0	--	0 (-0.7, 0.7); moderate[a]	0 (-0.7, 0.7); moderate
Tralokinumab	Placebo	3	-0.4 (-0.6, -0.3); high	--	-0.4 (-0.6, -0.3); high

Dose of dupilumab and tralokinumab is 600 mg then 300 mg every 2 weeks

© 2022 Drucker AM, et al. *JAMA Dermatology*.

The network meta-analysis certainty ratings start at the higher of the direct or indirect ratings and can then be rated down further for incoherence (based on the results of node splitting analyses) and imprecision.

* Rated down for significant issues related to inconsistency in head-to-head trials
a. Rated down for significant issues related to risk of bias in included trials
b. Rated down for imprecision

© 2022 Drucker AM, et al. *JAMA Dermatology*.

eFigure 3. Network graphs of studies included in the analysis of adults treated between 8 and 16 weeks for change in itch on the standardized mean difference scale. The width of each line connecting two treatments (nodes) is proportional to the number of head-to-head trials for that comparison.

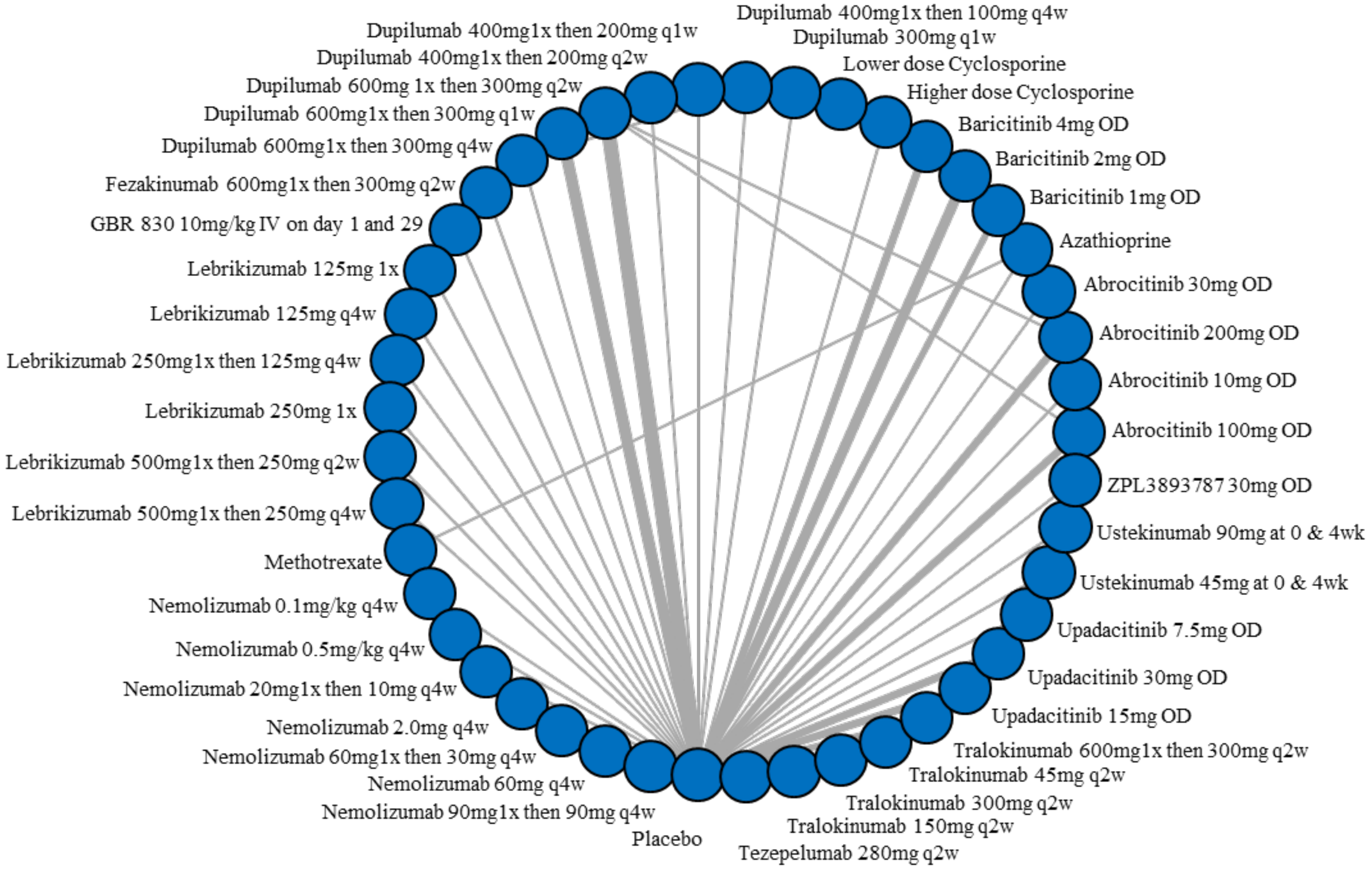

OD: once daily; q1w: once weekly; q2w: every 2 weeks; q4w: every 4 weeks.

© 2022 Drucker AM, et al. *JAMA Dermatology*.

eTable 14. League table with relative effect estimates for change in itch up to 16 weeks of treatment in adults for placebo and medications used in clinical practice or likely to be approved soon. Results for other pairwise comparisons in the network can be requested from the authors. Results are presented as change in standardized mean difference of itch scales (95% CrI). A positive effect estimate in a given cell favors the row-defining treatment. A negative effect estimate in a given cell favors the column-defining treatment. The bottom row contains the Surface Under the Cumulative Ranking (SUCRA) value for the column-defining treatment.

	Abrocitinib 100 mg daily	Abrocitinib 200 mg daily	Azathioprine	Baricitinib 2 mg daily	Baricitinib 4 mg daily	Cyclosporine (higher dose)	Cyclosporine (lower dose)	Dupilumab*	Methotrexate	Placebo	Tralokinumab*	Upadacitinib 15 mg daily	Upadacitinib 30 mg daily
Abrocitinib 100 mg daily		-0.3 (-0.5, -0.2)	0 (-0.7, 0.6)	0 (-0.2, 0.3)	-0.1 (-0.3, 0.2)	-0.2 (-1, 0.7)	-0.1 (-1.1, 0.8)	-0.3 (-0.5, -0.1)	0 (-0.9, 0.9)	0.5 (0.4, 0.7)	0.1 (-0.1, 0.3)	-0.2 (-0.4, 0)	-0.5 (-0.7, -0.3)
Abrocitinib 200 mg daily	0.3 (0.2, 0.5)		0.3 (-0.4, 0.9)	0.4 (0.1, 0.6)	0.2 (0, 0.5)	0.2 (-0.7, 1)	0.2 (-0.8, 1.1)	0 (-0.1, 0.2)	0.3 (-0.5, 1.2)	0.9 (0.7, 1)	0.4 (0.2, 0.6)	0.1 (-0.1, 0.4)	-0.2 (-0.4, 0.1)
Azathioprine	0 (-0.6, 0.7)	-0.3 (-0.9, 0.4)		0.1 (-0.5, 0.7)	0 (-0.7, 0.6)	-0.1 (-1.1, 1)	-0.1 (-1.2, 1.1)	-0.2 (-0.8, 0.4)	0 (-0.6, 0.7)	0.6 (0, 1.2)	0.1 (-0.5, 0.8)	-0.2 (-0.8, 0.5)	-0.4 (-1.1, 0.2)
Baricitinib 2 mg daily	0 (-0.3, 0.2)	-0.4 (-0.6, -0.1)	-0.1 (-0.7, 0.5)		-0.1 (-0.3, 0.1)	-0.2 (-1.1, 0.7)	-0.2 (-1.1, 0.8)	-0.3 (-0.5, -0.1)	0 (-0.9, 0.8)	0.5 (0.3, 0.7)	0.1 (-0.2, 0.3)	-0.2 (-0.5, 0)	-0.5 (-0.8, -0.3)
Baricitinib 4 mg daily	0.1 (-0.2, 0.3)	-0.2 (-0.5, 0)	0 (-0.6, 0.7)	0.1 (-0.1, 0.3)		-0.1 (-0.9, 0.8)	-0.1 (-1, 0.9)	-0.2 (-0.4, 0)	0.1 (-0.8, 1)	0.6 (0.4, 0.8)	0.2 (-0.1, 0.4)	-0.1 (-0.4, 0.1)	-0.4 (-0.7, -0.2)
Cyclosporine (higher dose)	0.2 (-0.7, 1)	-0.2 (-1, 0.7)	0.1 (-1, 1.1)	0.2 (-0.7, 1.1)	0.1 (-0.8, 0.9)		0 (-0.4, 0.4)	-0.1 (-1, 0.7)	0.2 (-1.1, 1.4)	0.7 (-0.2, 1.5)	0.3 (-0.6, 1.1)	0 (-0.9, 0.8)	-0.3 (-1.2, 0.5)
Cyclosporine (lower dose)	0.1 (-0.8, 1.1)	-0.2 (-1.1, 0.8)	0.1 (-1.1, 1.2)	0.2 (-0.8, 1.1)	0.1 (-0.9, 1)	0 (-0.4, 0.4)		-0.1 (-1.1, 0.8)	0.2 (-1.2, 1.4)	0.7 (-0.3, 1.6)	0.2 (-0.7, 1.2)	-0.1 (-1, 0.9)	-0.3 (-1.3, 0.6)
Dupilumab*	0.3 (0.1, 0.5)	0 (-0.2, 0.1)	0.2 (-0.4, 0.8)	0.3 (0.1, 0.5)	0.2 (0, 0.4)	0.1 (-0.7, 1)	0.1 (-0.8, 1.1)		0.3 (-0.6, 1.2)	0.8 (0.7, 0.9)	0.4 (0.2, 0.6)	0.1 (-0.1, 0.3)	-0.2 (-0.4, 0)
Methotrexate	0 (-0.9, 0.9)	-0.3 (-1.2, 0.5)	0 (-0.7, 0.6)	0 (-0.8, 0.9)	-0.1 (-1, 0.8)	-0.2 (-1.4, 1.1)	-0.2 (-1.4, 1.2)	-0.3 (-1.2, 0.6)		0.5 (-0.3, 1.4)	0.1 (-0.8, 1)	-0.2 (-1.1, 0.7)	-0.5 (-1.4, 0.4)
Placebo	-0.5 (-0.7, -0.4)	-0.9 (-1, -0.7)	-0.6 (-1.2, 0)	-0.5 (-0.7, -0.3)	-0.6 (-0.8, -0.4)	-0.7 (-1.5, 0.2)	-0.7 (-1.6, 0.3)	-0.8 (-0.9, -0.7)	-0.5 (-1.4, 0.3)		-0.4 (-0.6, -0.3)	-0.7 (-0.9, -0.6)	-1 (-1.2, -0.9)
Tralokinumab*	-0.1 (-0.3, 0.1)	-0.4 (-0.6, -0.2)	-0.1 (-0.8, 0.5)	-0.1 (-0.3, 0.2)	-0.2 (-0.4, 0.1)	-0.3 (-1.1, 0.6)	-0.2 (-1.2, 0.7)	-0.4 (-0.6, -0.2)	-0.1 (-1, 0.8)	0.4 (0.3, 0.6)		-0.3 (-0.5, -0.1)	-0.6 (-0.8, -0.4)
Upadacitinib 15 mg daily	0.2 (0, 0.4)	-0.1 (-0.4, 0.1)	0.2 (-0.5, 0.8)	0.2 (0, 0.5)	0.1 (-0.1, 0.4)	0 (-0.8, 0.9)	0.1 (-0.9, 1)	-0.1 (-0.3, 0.1)	0.2 (-0.7, 1.1)	0.7 (0.6, 0.9)	0.3 (0.1, 0.5)		-0.3 (-0.4, -0.1)
Upadacitinib 30 mg daily	0.5 (0.3, 0.7)	0.2 (-0.1, 0.4)	0.4 (-0.2, 1.1)	0.5 (0.3, 0.8)	0.4 (0.2, 0.7)	0.3 (-0.5, 1.2)	0.3 (-0.6, 1.3)	0.2 (0, 0.4)	0.5 (-0.4, 1.4)	1 (0.9, 1.2)	0.6 (0.4, 0.8)	0.3 (0.1, 0.4)	
SUCRA	0.46	0.73	0.50	0.43	0.53	0.58	0.56	0.69	0.47	0.12	0.38	0.62	0.83

*Dose of dupilumab and tralokinumab is 600 mg then 300 mg every 2 weeks

© 2022 Drucker AM, et al. *JAMA Dermatology*.

eTable 15. GRADE certainty ratings for change in the standardized mean difference of itch up to 16 weeks of treatment in adults for placebo and medications used in clinical practice or likely to be approved soon. Results are presented standardized mean difference (95% CrI). Negative effect estimates favor the treatment listed in the intervention column; positive effect estimates favor the comparator.

Intervention	Comparator	Head-to-head trials, n	Direct estimate (95% CrI); Certainty of Evidence	Indirect estimate (95% CrI); Certainty of Evidence	NMA Estimate (95% CrI); Certainty of Evidence
Abrocitinib 100 mg daily	Abrocitinib 200 mg daily	4	0.3 (0.2, 0.5); high	--	0.3 (0.2, 0.5); high
Abrocitinib 100 mg daily	Azathioprine	0	--	0 (-0.6, 0.7); moderate[a]	0 (-0.6, 0.7); moderate[b]
Abrocitinib 100 mg daily	Baricitinib 2 mg daily	0	--	0 (-0.3, 0.2); high	0 (-0.3, 0.2); high
Abrocitinib 100 mg daily	Baricitinib 4 mg daily	0	--	0.1 (-0.2, 0.3); high	0.1 (-0.2, 0.3); high
Abrocitinib 100 mg daily	Cyclosporine (higher dose)	0	--	0.2 (-0.7, 1); moderate[a]	0.2 (-0.7, 1); low[b]
Abrocitinib 100 mg daily	Cyclosporine (lower dose)	0	--	0.1 (-0.8, 1.1); moderate[a]	0.1 (-0.8, 1.1); low[b]
Abrocitinib 100 mg daily	Dupilumab	1	0.3 (0.1, 0.5); high	0.3 (0, 0.5); high	0.3 (0.1, 0.5); high
Abrocitinib 100 mg daily	Methotrexate	0	--	0 (-0.9, 0.9); moderate[a]	0 (-0.9, 0.9); low[b]
Abrocitinib 100 mg daily	Placebo	4	-0.5 (-0.7, -0.4); high	--	-0.5 (-0.7, -0.4); high
Abrocitinib 100 mg daily	Tralokinumab	0	--	-0.1 (-0.3, 0.1); high	-0.1 (-0.3, 0.1); high
Abrocitinib 100 mg daily	Upadacitinib 15 mg daily	0	--	0.2 (0, 0.4); high	0.2 (0, 0.4); moderate[b]
Abrocitinib 100 mg daily	Upadacitinib 30 mg daily	0	--	0.5 (0.3, 0.7); high	0.5 (0.3, 0.7); high
Abrocitinib 200 mg daily	Azathioprine	0	--	-0.3 (-0.9, 0.4); moderate[a]	-0.3 (-0.9, 0.4); low[b]
Abrocitinib 200 mg daily	Baricitinib 2 mg daily	0	--	-0.4 (-0.6, -0.1); high	-0.4 (-0.6, -0.1); high
Abrocitinib 200 mg daily	Baricitinib 4 mg daily	0	--	-0.2 (-0.5, 0); high	-0.2 (-0.5, 0); moderate[b]
Abrocitinib 200 mg daily	Cyclosporine (higher dose)	0	--	-0.2 (-1, 0.7); moderate[a]	-0.2 (-1, 0.7); low[b]

© 2022 Drucker AM, et al. *JAMA Dermatology*.

Abrocitinib 200 mg daily	Cyclosporine (lower dose)	0	--	-0.2 (-1.1, 0.8); moderate[a]	-0.2 (-1.1, 0.8); low[b]
Abrocitinib 200 mg daily	Dupilumab	1	-0.1 (-0.4, 0.1); high	0 (-0.2, 0.3); high	0 (-0.2, 0.1); high
Abrocitinib 200 mg daily	Methotrexate	0	--	-0.3 (-1.2, 0.5); moderate[a]	-0.3 (-1.2, 0.5); low[b]
Abrocitinib 200 mg daily	Placebo	4	-0.9 (-1, -0.7); high	--	-0.9 (-1, -0.7); high
Abrocitinib 200 mg daily	Tralokinumab	0	--	-0.4 (-0.6, -0.2); high	-0.4 (-0.6, -0.2); high
Abrocitinib 200 mg daily	Upadacitinib 15 mg daily	0	--	-0.1 (-0.4, 0.1; high	-0.1 (-0.4, 0.1); high
Abrocitinib 200 mg daily	Upadacitinib 30 mg daily	0	--	0.2 (-0.1, 0.4); high	0.2 (-0.1, 0.4); moderate[b]
Azathioprine	Baricitinib 2 mg daily	0	--	-0.1 (-0.7, 0.5); moderate[a]	-0.1 (-0.7, 0.5); moderate
Azathioprine	Baricitinib 4 mg daily	0	--	0 (-0.6, 0.7); moderate[a]	0 (-0.6, 0.7); moderate
Azathioprine	Cyclosporine (higher dose)	0	--	0.1 (-1, 1.1); moderate[a]	0.1 (-1, 1.1); low[b]
Azathioprine	Cyclosporine (lower dose)	0	--	0.1 (-1.1, 1.2); moderate[a]	0.1 (-1.1, 1.2); low[b]
Azathioprine	Dupilumab	0	--	0.2 (-0.4, 0.8); moderate[a]	0.2 (-0.4, 0.8); low[b]
Azathioprine	Methotrexate	1	0 (-0.7, 0.6); moderate[a]	--	0 (-0.7, 0.6); moderate
Azathioprine	Placebo	1	-0.6 (-1.2, 0); moderate[a]	--	-0.6 (-1.2, 0); low[b]
Azathioprine	Tralokinumab	0	--	-0.1 (-0.8, 0.5); moderate[a]	-0.1 (-0.8, 0.5); low[b]
Azathioprine	Upadacitinib 15 mg daily	0	--	0.2 (-0.5, 0.8); moderate[a]	0.2 (-0.5, 0.8); low[b]
Azathioprine	Upadacitinib 30 mg daily	0	--	0.4 (-0.2, 1.1); moderate[a]	0.4 (-0.2, 1.1); low[b]
Baricitinib 2 mg daily	Baricitinib 4 mg daily	4	0.1 (-0.1, 0.3); high	--	0.1 (-0.1, 0.3); high
Baricitinib 2 mg daily	Cyclosporine (higher dose)	0	--	0.2 (-0.7, 1.1); moderate[a]	0.2 (-0.7, 1.1); low[b]
Baricitinib 2 mg daily	Cyclosporine (lower dose)	0	--	0.2 (-0.8, 1.1); moderate[a]	0.2 (-0.8, 1.1); low[b]
Baricitinib 2 mg daily	Dupilumab	0	--	0.3 (0.1, 0.5); high	0.3 (0.1, 0.5); high

© 2022 Drucker AM, et al. *JAMA Dermatology*.

Baricitinib 2 mg daily	Methotrexate	0	--	0 (-0.8, 0.9); moderate[a]	0 (-0.8, 0.9); low[b]
Baricitinib 2 mg daily	Placebo	5	-0.5 (-0.7, -0.3); high	--	-0.5 (-0.7, -0.3); high
Baricitinib 2 mg daily	Tralokinumab	0	--	-0.1 (-0.3, 0.2); high	-0.1 (-0.3, 0.2); high
Baricitinib 2 mg daily	Upadacitinib 15 mg daily	0	--	0.2 (0, 0.5); high	0.2 (0, 0.5); moderate[b]
Baricitinib 2 mg daily	Upadacitinib 30 mg daily	0	--	0.5 (0.3, 0.8); high	0.5 (0.3, 0.8); high
Baricitinib 4 mg daily	Cyclosporine (higher dose)	0	--	0.1 (-0.8, 0.9); moderate[a]	0.1 (-0.8, 0.9); low[b]
Baricitinib 4 mg daily	Cyclosporine (lower dose)	0	--	0.1 (-0.9, 1); moderate[a]	0.1 (-0.9, 1); low[b]
Baricitinib 4 mg daily	Dupilumab	0	--	0.2 (0, 0.4); high	0.2 (0, 0.4); moderate[b]
Baricitinib 4 mg daily	Methotrexate	0	--	-0.1 (-1, 0.8); moderate[a]	-0.1 (-1, 0.8); low[b]
Baricitinib 4 mg daily	Placebo	4	-0.6 (-0.8, -0.4); high	--	-0.6 (-0.8, -0.4); high
Baricitinib 4 mg daily	Tralokinumab	0	--	-0.2 (-0.4, 0.1); high	-0.2 (-0.4, 0.1); moderate[b]
Baricitinib 4 mg daily	Upadacitinib 15 mg daily	0	--	0.1 (-0.1, 0.4); high	0.1 (-0.1, 0.4); high
Baricitinib 4 mg daily	Upadacitinib 30 mg daily	0	--	0.4 (0.2, 0.7); high	0.4 (0.2, 0.7); high
Cyclosporine (higher dose)	Cyclosporine (lower dose)	1	0 (-0.4, 0.4); moderate[a]	--	0 (-0.4, 0.4); moderate
Cyclosporine (higher dose)	Dupilumab	0	--	0.1 (-0.7, 1); moderate[a]	0.1 (-0.7, 1); low[b]
Cyclosporine (higher dose)	Methotrexate	0	--	-0.2 (-1.4, 1.1); moderate[a]	-0.2 (-1.4, 1.1); low[b]
Cyclosporine (higher dose)	Placebo	1	-0.7 (-1.5, 0.2); moderate[a]	--	-0.7 (-1.5, 0.2); low[b]
Cyclosporine (higher dose)	Tralokinumab	0	--	-0.3 (-1.1, 0.6); moderate[a]	-0.3 (-1.1, 0.6); low[b]
Cyclosporine (higher dose)	Upadacitinib 15 mg daily	0	--	0 (-0.8, 0.9); moderate[a]	0 (-0.8, 0.9); low[b]
Cyclosporine (higher dose)	Upadacitinib 30 mg daily	0	--	0.3 (-0.5, 1.2); moderate[a]	0.3 (-0.5, 1.2); low[b]
Cyclosporine (lower dose)	Dupilumab	0	--	0.1 (-0.8, 1.1); moderate[a]	0.1 (-0.8, 1.1); low[b]

© 2022 Drucker AM, et al. *JAMA Dermatology*.

Cyclosporine (lower dose)	Methotrexate	0	-	-0.2 (-1.4, 1.2); moderate[a]	-0.2 (-1.4, 1.2); low[b]
Cyclosporine (lower dose)	Placebo	0	--	-0.7 (-1.6, 0.3); moderate[a]	-0.7 (-1.6, 0.3); low[b]
Cyclosporine (lower dose)	Tralokinumab	0	--	-0.2 (-1.2, 0.7); moderate[a]	-0.2 (-1.2, 0.7); low[b]
Cyclosporine (lower dose)	Upadacitinib 15 mg daily	0	--	0.1 (-0.9, 1); moderate[a]	0.1 (-0.9, 1); low[b]
Cyclosporine (lower dose)	Upadacitinib 30 mg daily	0	--	0.3 (-0.6, 1.3); moderate[a]	0.3 (-0.6, 1.3); low[b]
Dupilumab	Methotrexate	0	--	-0.3 (-1.2, 0.6); moderate[a]	-0.3 (-1.2, 0.6); low[b]
Dupilumab	Placebo	7	-0.8 (-0.9, -0.7); high	--	-0.8 (-0.9, -0.7); high
Dupilumab	Tralokinumab	0	--	-0.4 (-0.6, -0.2); high	-0.4 (-0.6, -0.2); high
Dupilumab	Upadacitinib 15 mg daily	0	--	-0.1 (-0.3, 0.1); high	-0.1 (-0.3, 0.1); high
Dupilumab	Upadacitinib 30 mg daily	0	--	0.2 (0, 0.4); high	0.2 (0, 0.4); moderate[b]
Methotrexate	Placebo	0	--	-0.5 (-1.4, 0.3); moderate[a]	-0.5 (-1.4, 0.3); low[b]
Methotrexate	Tralokinumab	0	--	-0.1 (-1, 0.8); moderate[a]	-0.1 (-1, 0.8); low[b]
Methotrexate	Upadacitinib 15 mg daily	0	--	0.2 (-0.7, 1.1); moderate[a]	0.2 (-0.7, 1.1); low[b]
Methotrexate	Upadacitinib 30 mg daily	0	--	0.5 (-0.4, 1.4); moderate[a]	0.5 (-0.4, 1.4); low[b]
Tralokinumab	Placebo	3		--	-0.4 (-0.6, -0.3); high
Tralokinumab	Upadacitinib 15 mg daily	0	--	0.3 (0.1, 0.5); high	0.3 (0.1, 0.5); high
Tralokinumab	Upadacitinib 30 mg daily	0	--	0.6 (0.4, 0.8); high	0.6 (0.4, 0.8); high
Upadacitinib 15 mg daily	Upadacitinib 30 mg daily	4	0.3 (0.1, 0.4); high	--	0.3 (0.1, 0.4); high
Upadacitinib 15 mg daily	Placebo	4	0.7 (0.6, 0.9); high	--	0.7 (0.6, 0.9); high
Upadacitinib 30 mg daily	Placebo	4	1 (0.9, 1.2); high	--	1 (0.9, 1.2) high

Dose of dupilumab and tralokinumab is 600 mg then 300 mg every 2 weeks

© 2022 Drucker AM, et al. *JAMA Dermatology*.

The network meta-analysis certainty ratings start at the higher of the direct or indirect ratings and can then be rated down further for incoherence (based on the results of node splitting analyses) and imprecision.

a. Rated down for significant issues related to risk of bias in included trials
b. Rated down for imprecision

© 2022 Drucker AM, et al. *JAMA Dermatology*.

eFigure 4. Network graphs of studies included in the analysis of adults treated between 8 and 16 weeks for withdrawal due to adverse events. The width of each line connecting two treatments (nodes) is proportional to the number of head-to-head trials for that comparison.

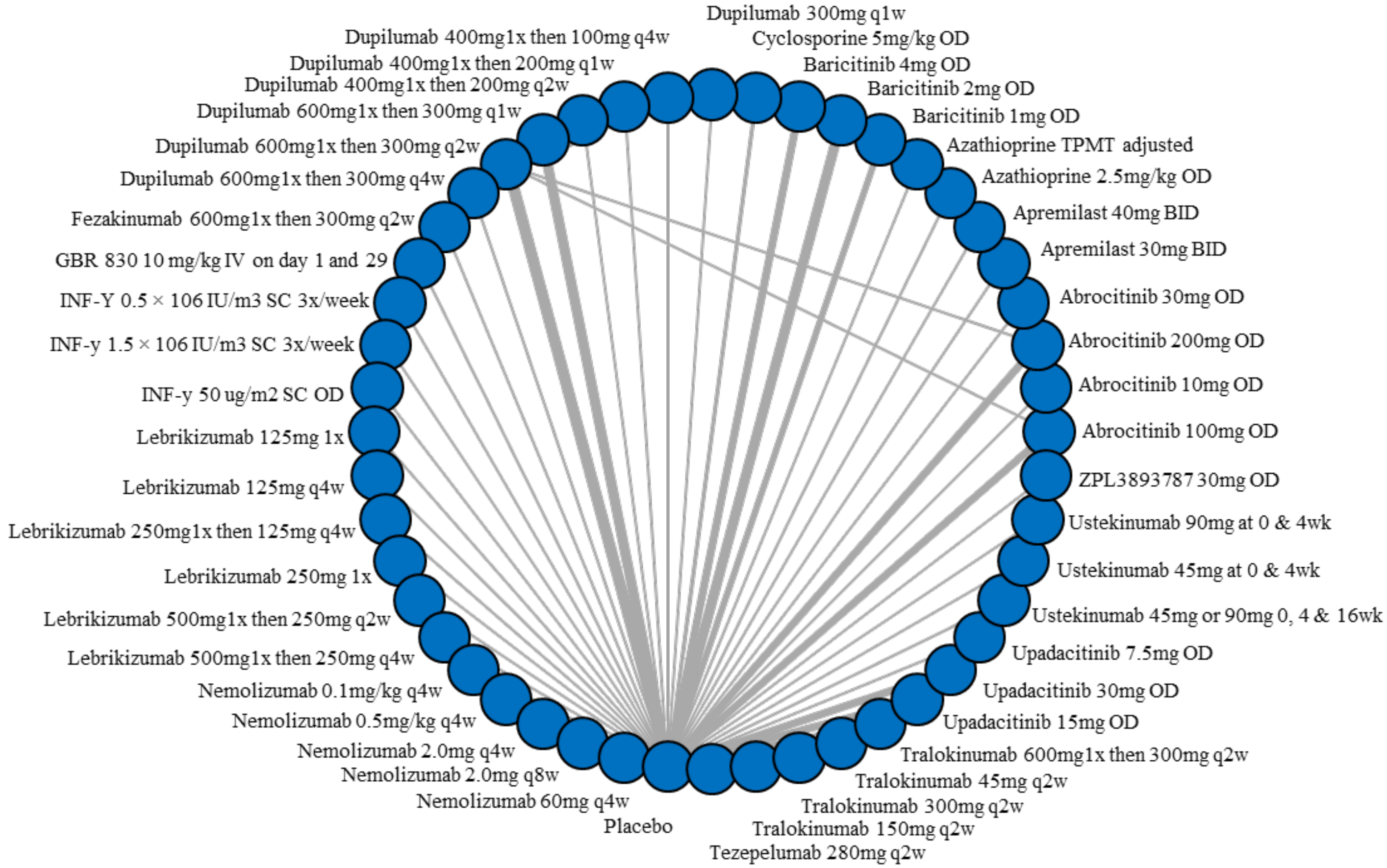

OD: once daily; q1w: once weekly; q2w: every 2 weeks; q4w: every 4 weeks.

© 2022 Drucker AM, et al. *JAMA Dermatology*.

eTable 16. League table with relative effect estimates for withdrawals from adverse events up to 16 weeks of treatment in adults for placebo and medications used in clinical practice or likely to be approved soon. Results for other pairwise comparisons in the network can be requested from the authors. Results are presented as Odds Ratios (95% CrI). Odds ratios are given as the odds with the column-defining treatment over the odds with the row-defining treatment. Odds ratios over 1 indicate more withdrawals due to serious adverse event with the intervention vs. comparator.

	Abrocitinib 100 mg daily	Abrocitinib 200 mg daily	Baricitinib 2 mg daily	Baricitinib 4 mg daily	Dupilumab 600 mg then 300 mg q2 weeks	Placebo	Tralokinumab 600 mg then 300 mg q2 weeks	Upadacitinib 15 mg daily	Upadacitinib 30 mg daily
Abrocitinib 100 mg daily		1 (0.5, 1.7)	1.1 (0.4, 3)	2.1 (0.8, 5.5)	1.3 (0.6, 2.8)	1.3 (0.8, 2.4)	1.2 (0.5, 2.9)	0.8 (0.3, 1.8)	1 (0.4, 2.2)
Abrocitinib 200 mg daily	1.1 (0.6, 1.8)		1.2 (0.4, 3.1)	2.2 (0.8, 5.9)	1.3 (0.6, 2.9)	1.4 (0.8, 2.5)	1.3 (0.5, 3.1)	0.8 (0.3, 1.9)	1 (0.5, 2.3)
Baricitinib 2 mg daily	0.9 (0.3, 2.5)	0.9 (0.3, 2.4)		1.9 (0.8, 4.8)	1.2 (0.4, 3.4)	1.2 (0.5, 2.9)	1.1 (0.4, 3.2)	0.7 (0.3, 2)	0.9 (0.3, 2.5)
Baricitinib 4 mg daily	0.5 (0.2, 1.3)	0.5 (0.2, 1.3)	0.5 (0.2, 1.3)		0.6 (0.2, 1.7)	0.7 (0.3, 1.5)	0.6 (0.2, 1.6)	0.4 (0.1, 1)	0.5 (0.2, 1.3)
Dupilumab 600 mg then 300 mg q2 weeks	0.8 (0.4, 1.7)	0.7 (0.3, 1.6)	0.9 (0.3, 2.4)	1.6 (0.6, 4.5)		1 (0.6, 2)	0.9 (0.4, 2.4)	0.6 (0.3, 1.4)	0.8 (0.3, 1.8)
Placebo	0.7 (0.4, 1.3)	0.7 (0.4, 1.3)	0.8 (0.3, 1.9)	1.5 (0.7, 3.4)	1 (0.5, 1.8)		0.9 (0.5, 1.8)	0.6 (0.3, 1)	0.7 (0.4, 1.3)
Tralokinumab 600 mg then 300 mg q2 weeks	0.8 (0.3, 2)	0.8 (0.3, 1.9)	0.9 (0.3, 2.6)	1.7 (0.6, 4.7)	1.1 (0.4, 2.6)	1.1 (0.6, 2.1)		0.6 (0.3, 1.5)	0.8 (0.3, 1.9)
Upadacitinib 15 mg daily	1.3 (0.6, 3)	1.2 (0.5, 2.9)	1.4 (0.5, 3.9)	2.7 (1, 7.4)	1.7 (0.7, 4)	1.7 (1, 3.2)	1.6 (0.7, 3.9)		1.3 (0.7, 2.4)
Upadacitinib 30 mg daily	1 (0.5, 2.3)	1 (0.4, 2.2)	1.1 (0.4, 3.1)	2.1 (0.8, 5.7)	1.3 (0.6, 3.1)	1.4 (0.8, 2.5)	1.2 (0.5, 3)	0.8 (0.4, 1.5)	
SUCRA	0.69	0.71	0.66	0.45	0.60	0.58	0.62	0.77	0.70

© 2022 Drucker AM, et al. *JAMA Dermatology*.

eTable 17. GRADE certainty ratings for withdrawals from adverse events up to 16 weeks of treatment in adults for placebo and medications used in clinical practice or likely to be approved soon. Results are presented as Odds Ratios (95% CrI). Odds ratios are given as the odds with the treatment in the intervention column over the odds of the treatment in the comparator column. Odds ratios over 1 indicate more withdrawals due to serious adverse event with the intervention vs. comparator.

Intervention	Comparator	Head-to-head trials, n	Direct estimate (95% CrI); Certainty of Evidence	Indirect estimate (95% CrI); Certainty of Evidence	NMA Estimate (95% CrI); Certainty of Evidence
Abrocitinib 100 mg daily	Abrocitinib 200 mg daily	4	1.1 (0.6, 1.8); moderate*	--	1.1 (0.6, 1.8); low[a]
Abrocitinib 100 mg daily	Baricitinib 2 mg daily	0	--	0.9 (0.3, 2.5); moderate*	0.9 (0.3, 2.5); very low[b]
Abrocitinib 100 mg daily	Baricitinib 4 mg daily	0	--	0.5 (0.2, 1.3); moderate*	0.5 (0.2, 1.3); very low[b]
Abrocitinib 100 mg daily	Dupilumab	1	0.8 (0.2, 3.6); high	0.6 (0.2, 1.8); moderate*	0.8 (0.4, 1.7); low [a]
Abrocitinib 100 mg daily	Placebo	4	0.7 (0.4, 1.3); moderate*	--	0.7 (0.4, 1.3); low [a]
Abrocitinib 100 mg daily	Tralokinumab	0	--	0.8 (0.3, 2); moderate*	0.8 (0.3, 2); very low[b]
Abrocitinib 100 mg daily	Upadacitinib 15 mg daily	0	--	1.3 (0.6, 3); moderate*	1.3 (0.6, 3); very low[b]
Abrocitinib 100 mg daily	Upadacitinib 30 mg daily	0	--	1 (0.5, 2.3); moderate*	1 (0.5, 2.3); very low[b]
Abrocitinib 200 mg daily	Baricitinib 2 mg daily	0	--	0.9 (0.3, 2.4); moderate*	0.9 (0.3, 2.4); very low[b]
Abrocitinib 200 mg daily	Baricitinib 4 mg daily	0	--	0.5 (0.2, 1.3); moderate*	0.5 (0.2, 1.3); very low[b]
Abrocitinib 200 mg daily	Dupilumab	1	1.5 (0.4, 5.9); high	0.4 (0.1, 1.4); moderate*	0.8 (0.3, 1.6); very low[b]
Abrocitinib 200 mg daily	Placebo	4	0.7 (0.4, 1.3); moderate*	--	0.7 (0.4, 1.3); low [a]
Abrocitinib 200 mg daily	Tralokinumab	0	--	0.8 (0.3, 1.9); moderate*	0.8 (0.3, 1.9); very low[b]

© 2022 Drucker AM, et al. *JAMA Dermatology*.

Abrocitinib 200 mg daily	Upadacitinib 15 mg daily	0	--	1.2 (0.5, 2.9); moderate*	1.2 (0.5, 2.9); very low[b]
Abrocitinib 200 mg daily	Upadacitinib 30 mg daily	0	--	1 (0.4, 2.2); moderate*	1 (0.4, 2.2); very low[b]
Baricitinib 2 mg daily	Baricitinib 4 mg daily	4	0.5 (0.2, 1.3); moderate*	--	0.5 (0.2, 1.3); low [a]
Baricitinib 2 mg daily	Dupilumab	0	--	0.9 (0.3, 2.4); moderate*	0.9 (0.3, 2.4); very low[b]
Baricitinib 2 mg daily	Placebo	5	0.8 (0.3, 1.9); moderate*	--	0.8 (0.3, 1.9); very low[b]
Baricitinib 2 mg daily	Tralokinumab	0	--	0.9 (0.3, 2.6); moderate*	0.9 (0.3, 2.6); very low[b]
Baricitinib 2 mg daily	Upadacitinib 15 mg daily	0	--	1.4 (0.5, 3.9); moderate*	1.4 (0.5, 3.9); very low[b]
Baricitinib 2 mg daily	Upadacitinib 30 mg daily	0	--	1.1 (0.4, 3.1); moderate*	1.1 (0.4, 3.1); very low[b]
Baricitinib 4 mg daily	Dupilumab	0	--	1.6 (0.6, 4.5); moderate*	1.6 (0.6, 4.5); very low[b]
Baricitinib 4 mg daily	Placebo	4	1.5 (0.7, 3.4); moderate*	--	1.5 (0.7, 3.4); very low[b]
Baricitinib 4 mg daily	Tralokinumab	0	--	1.7 (0.6, 4.7); moderate*	1.7 (0.6, 4.7); very low[b]
Baricitinib 4 mg daily	Upadacitinib 15 mg daily	0	--	2.7 (1, 7.4); moderate*	2.7 (1, 7.4); very low[b]
Baricitinib 4 mg daily	Upadacitinib 30 mg daily	0	--	2.1 (0.8, 5.7); moderate*	2.1 (0.8, 5.7); very low[b]
Dupilumab	Placebo	6	1 (0.5, 1.8); moderate*	--	1 (0.5, 1.8); low[a]
Dupilumab	Tralokinumab	0	--	1.1 (0.4, 2.6); moderate*	1.1 (0.4, 2.6); very low[b]
Dupilumab	Upadacitinib 15 mg daily	0	--	1.7 (0.7, 4); moderate*	1.7 (0.7, 4); very low[b]
Dupilumab	Upadacitinib 30 mg daily	0	--	1.3 (0.6, 3.1); moderate*	1.3 (0.6, 3.1); very low[b]
Tralokinumab	Upadacitinib 15 mg daily	0	--	1.6 (0.7, 3.9); moderate*	1.6 (0.7, 3.9); very low[b]

© 2022 Drucker AM, et al. *JAMA Dermatology*.

Tralokinumab	Upadacitinib 30 mg daily	0	--	1.2 (0.5, 3); moderate*	1.2 (0.5, 3); very low[b]
Tralokinumab	Placebo	4	0.9 (0.5, 1.8); moderate*	--	0.9 (0.5, 1.8); low [a]
Upadacitinib 15 mg daily	Upadacitinib 30 mg daily	4	0.8 (0.4, 1.5); moderate*	--	0.8 (0.4, 1.5); low [a]
Upadacitinib 15 mg daily	Placebo	4	0.6 (0.3, 1); high	--	0.6 (0.3, 1); moderate [a]
Upadacitinib 30 mg daily	Placebo	4	0.7 (0.4, 1.3); moderate*	--	0.7 (0.4, 1.3); low [a]

Dose of dupilumab and tralokinumab is 600 mg then 300 mg every 2 weeks

The network meta-analysis certainty ratings start at the higher of the direct or indirect ratings and can then be rated down further for incoherence (based on the results of node splitting analyses) and imprecision.

* Rated down for inconsistency
a. Rated down for imprecision (estimate imprecise and would suggest different conclusions at either end of the 95% Credible Intervals)
b. Rated down 2 levels for imprecision (estimate imprecise and would suggest different conclusions at either end of the 95% Credible Intervals and very wide credible intervals

© 2022 Drucker AM, et al. *JAMA Dermatology*.

eFigure 5. Network graphs of studies included in the analysis of adults treated between 8 and 16 weeks for serious adverse events. The width of each line connecting two treatments (nodes) is proportional to the number of head-to-head trials for that comparison.

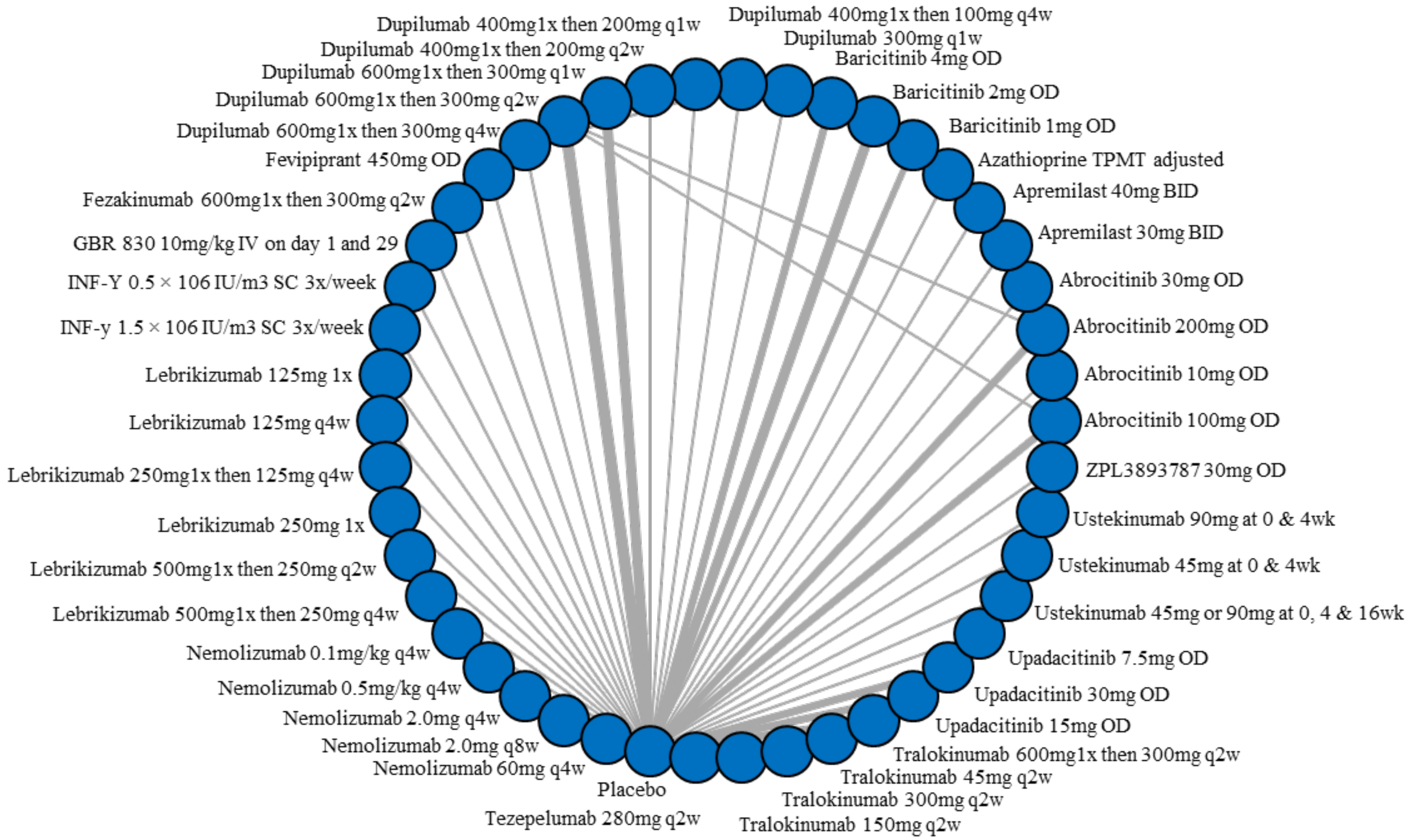

OD: once daily; q1w: once weekly; q2w: every 2 weeks; q4w: every 4 weeks.

© 2022 Drucker AM, et al. *JAMA Dermatology*.

eTable 18. League table with relative effect estimates for serious adverse events up to 16 weeks of treatment in adults for placebo and medications used in clinical practice or likely to be approved soon. Results for other pairwise comparisons in the network can be requested from the authors. Results are presented as Odds Ratios (95% CrI). Odds ratios are given as the odds with the column-defining treatment over the odds with the row-defining treatment. Odds ratios over 1 indicate more adverse serious adverse event with the intervention vs. comparator.

	Abrocitinib 100 mg daily	Abrocitinib 200 mg daily	Baricitinib 2 mg daily	Baricitinib 4 mg daily	Dupilumab 600 mg then 300 mg q2 weeks	Placebo	Tralokinumab 600 mg then 300 mg q2 weeks	Upadacitinib 15 mg daily	Upadacitinib 30 mg daily
Abrocitinib 100 mg daily		0.5 (0.2, 1.1)	0.4 (0.1, 1.1)	0.6 (0.2, 1.7)	0.4 (0.2, 0.9)	0.8 (0.4, 1.7)	0.6 (0.2, 1.5)	0.6 (0.2, 1.5)	0.6 (0.2, 1.5)
Abrocitinib 200 mg daily	1.9 (0.9, 4.2)		0.7 (0.2, 2.2)	1.1 (0.3, 3.4)	0.7 (0.3, 1.9)	1.5 (0.7, 3.7)	1.1 (0.4, 3.1)	1.1 (0.4, 3)	1 (0.4, 3)
Baricitinib 2 mg daily	2.7 (0.9, 8.3)	1.4 (0.5, 4.6)		1.6 (0.6, 4)	1 (0.4, 2.8)	2.2 (1, 5)	1.5 (0.6, 4.3)	1.5 (0.6, 4.2)	1.5 (0.5, 4.1)
Baricitinib 4 mg daily	1.7 (0.6, 5.1)	0.9 (0.3, 2.9)	0.6 (0.3, 1.6)		0.7 (0.3, 1.7)	1.4 (0.7, 3.1)	1 (0.4, 2.6)	1 (0.4, 2.6)	0.9 (0.4, 2.5)
Dupilumab 600 mg then 300 mg q2 weeks	2.6 (1.1, 6.4)	1.4 (0.5, 3.6)	1 (0.4, 2.5)	1.5 (0.6, 3.9)		2.1 (1.2, 3.9)	1.5 (0.6, 3.5)	1.4 (0.6, 3.4)	1.4 (0.6, 3.4)
Placebo	1.2 (0.6, 2.6)	0.6 (0.3, 1.5)	0.5 (0.2, 1)	0.7 (0.3, 1.4)	0.5 (0.3, 0.8)		0.7 (0.4, 1.3)	0.7 (0.4, 1.3)	0.7 (0.3, 1.2)
Tralokinumab 600 mg then 300 mg q2 weeks	1.7 (0.7, 4.7)	0.9 (0.3, 2.7)	0.7 (0.2, 1.7)	1 (0.4, 2.6)	0.7 (0.3, 1.6)	1.4 (0.8, 2.7)		1 (0.4, 2.3)	1 (0.4, 2.3)
Upadacitinib 15 mg daily	1.8 (0.7, 4.8)	0.9 (0.3, 2.7)	0.7 (0.2, 1.8)	1 (0.4, 2.7)	0.7 (0.3, 1.6)	1.5 (0.8, 2.8)	1 (0.4, 2.5)		1 (0.5, 2)
Upadacitinib 30 mg daily	1.8 (0.7, 5)	1 (0.3, 2.8)	0.7 (0.2, 1.8)	1.1 (0.4, 2.8)	0.7 (0.3, 1.7)	1.5 (0.8, 2.9)	1 (0.4, 2.6)	1 (0.5, 2)	
SUCRA	0.37	0.56	0.67	0.54	0.66	0.42	0.54	0.55	0.56

© 2022 Drucker AM, et al. *JAMA Dermatology*.

eTable 19. GRADE certainty ratings for serious adverse events up to 16 weeks of treatment in adults for placebo and medications used in clinical practice or likely to be approved soon. Results are presented as Odds Ratios (95% CrI). Odds ratios are given as the odds with the treatment in the intervention column over the odds of the treatment in the comparator column. Odds ratios over 1 indicate more adverse serious adverse event with the intervention vs. comparator.

Intervention	Comparator	Head-to-head trials, n	Direct estimate (95% CrI); Certainty of Evidence	Indirect estimate (95% CrI); Certainty of Evidence	NMA Estimate (95% CrI); Certainty of Evidence
Abrocitinib 100 mg daily	Abrocitinib 200 mg daily	4	1.9 (0.9, 4.2); moderate*	--	1.9 (0.9, 4.2); very low[c]
Abrocitinib 100 mg daily	Baricitinib 2 mg daily	0	--	2.7 (0.9, 8.3); moderate*	2.7 (0.9, 8.3); very low[c]
Abrocitinib 100 mg daily	Baricitinib 4 mg daily	0	--	1.7 (0.6, 5.1); moderate*	1.7 (0.6, 5.1); very low[c]
Abrocitinib 100 mg daily	Dupilumab	1	3.4 (0.6, 27.8); high	2.2 (0.7, 7.7); moderate*	2.6 (1.1, 6.4); low[b]
Abrocitinib 100 mg daily	Placebo	4	1.2 (0.6, 2.6); moderate*	--	1.2 (0.6, 2.6); very low[c]
Abrocitinib 100 mg daily	Tralokinumab	0	--	1.7 (0.7, 4.7); moderate*	1.7 (0.7, 4.7); very low[c]
Abrocitinib 100 mg daily	Upadacitinib 15 mg daily	0	--	1.8 (0.7, 4.8); moderate*	1.8 (0.7, 4.8); very low[c]
Abrocitinib 100 mg daily	Upadacitinib 30 mg daily	0	--	1.8 (0.7, 5); moderate*	1.8 (0.7, 5); very low[c]
Abrocitinib 200 mg daily	Baricitinib 2 mg daily	0	--	1.4 (0.5, 4.6); moderate*	1.4 (0.5, 4.6); very low[c]
Abrocitinib 200 mg daily	Baricitinib 4 mg daily	0	--	0.9 (0.3, 2.9); moderate*	0.9 (0.3, 2.9); very low[c]
Abrocitinib 200 mg daily	Dupilumab	1	1.1 (0.1, 12.2); high	1.6 (0.5, 5.6); moderate*	1.4 (0.5, 3.6); very low[c]
Abrocitinib 200 mg daily	Placebo	4	0.6 (0.3, 1.5); moderate*	--	0.6 (0.3, 1.5); very low[c]
Abrocitinib 200 mg daily	Tralokinumab	0	--	0.9 (0.3, 2.7); moderate*	0.9 (0.3, 2.7); very low[c]

© 2022 Drucker AM, et al. *JAMA Dermatology*.

Abrocitinib 200 mg daily	Upadacitinib 15 mg daily	0	--	0.9 (0.3, 2.7); moderate*	0.9 (0.3, 2.7); very low[c]
Abrocitinib 200 mg daily	Upadacitinib 30 mg daily	0	--	1 (0.3, 2.8); moderate*	1 (0.3, 2.8); very low[c]
Baricitinib 2 mg daily	Baricitinib 4 mg daily	4	0.6 (0.3, 1.6); moderate*	--	0.6 (0.3, 1.6); low[a]
Baricitinib 2 mg daily	Dupilumab	0	--	1 (0.4, 2.5); moderate*	1 (0.4, 2.5); very low[c]
Baricitinib 2 mg daily	Placebo	5	0.5 (0.2, 1); high	--	0.5 (0.2, 1); low[a]
Baricitinib 2 mg daily	Tralokinumab	0	--	0.7 (0.2, 1.7); moderate*	0.7 (0.2, 1.7); very low[c]
Baricitinib 2 mg daily	Upadacitinib 15 mg daily	0	--	0.7 (0.2, 1.8); high	0.7 (0.2, 1.8); low[c]
Baricitinib 2 mg daily	Upadacitinib 30 mg daily	0	--	0.7 (0.2, 1.8); high	0.7 (0.2, 1.8); low[c]
Baricitinib 4 mg daily	Dupilumab	0	--	1.5 (0.6, 3.9); moderate*	1.5 (0.6, 3.9); very low[c]
Baricitinib 4 mg daily	Placebo	4	0.7 (0.3, 1.4); moderate*	--	0.7 (0.3, 1.4); low[a]
Baricitinib 4 mg daily	Tralokinumab	0	--	1 (0.4, 2.6); moderate*	1 (0.4, 2.6); very low[c]
Baricitinib 4 mg daily	Upadacitinib 15 mg daily	0	--	1 (0.4, 2.7); moderate*	1 (0.4, 2.7); very low[c]
Baricitinib 4 mg daily	Upadacitinib 30 mg daily	0	--	1.1 (0.4, 2.8); moderate*	1.1 (0.4, 2.8); very low[c]
Dupilumab	Placebo	6	0.5 (0.3, 0.8); moderate*	--	0.5 (0.3, 0.8); moderate
Dupilumab	Tralokinumab	0	--	0.7 (0.3, 1.6); moderate*	0.7 (0.3, 1.6); very low[c]
Dupilumab	Upadacitinib 15 mg daily	0	--	0.7 (0.3, 1.6); moderate*	0.7 (0.3, 1.6); very low[c]
Dupilumab	Upadacitinib 30 mg daily	0	--	0.7 (0.3, 1.7); moderate*	0.7 (0.3, 1.7); very low[c]
Tralokinumab	Upadacitinib 15 mg daily	0	--	1 (0.4, 2.5); moderate*	1 (0.4, 2.5); very low[c]
Tralokinumab	Upadacitinib 30 mg daily	0	--	1 (0.4, 2.6); moderate*	1 (0.4, 2.6); very low[c]

© 2022 Drucker AM, et al. *JAMA Dermatology*.

Tralokinumab	Placebo	4	0.7 (0.4, 1.3); moderate*	--	0.7 (0.4, 1.3); low[a]
Upadacitinib 15 mg daily	Upadacitinib 30 mg daily	4	1 (0.5, 2); moderate*	--	1 (0.5, 2); very low[c]
Upadacitinib 15 mg daily	Placebo	4	0.7 (0.4, 1.3); high	--	0.7 (0.4, 1.3); moderate[a]
Upadacitinib 30 mg daily	Placebo	4	0.7 (0.3, 1.2); high	--	0.7 (0.3, 1.2); moderate[a]

Dose of dupilumab and tralokinumab is 600 mg then 300 mg every 2 weeks

The network meta-analysis certainty ratings start at the higher of the direct or indirect ratings and can then be rated down further for incoherence (based on the results of node splitting analyses) and imprecision.

* Rated down for inconsistency
a. Rated down for imprecision (estimate imprecise and would suggest different conclusions at either end of the 95% Credible Intervals)
b. Rated down for imprecision (very wide credible intervals)
c. Rated down 2 levels for imprecision (estimate imprecise and would suggest different conclusions at either end of the 95% Credible Intervals and very wide credible intervals)

© 2022 Drucker AM, et al. *JAMA Dermatology*.

<u>**Supplement references**</u>

1. Chan S, Cornelius V, Cro S, Harper JI, Lack G. Treatment Effect of Omalizumab on Severe Pediatric Atopic Dermatitis: The ADAPT Randomized Clinical Trial. *JAMA Pediatr.* 2019.
2. Simpson EL, Lacour JP, Spelman L, et al. Baricitinib in patients with moderate-to-severe atopic dermatitis and inadequate response to topical corticosteroids: results from two randomized monotherapy phase III trials. *The British journal of dermatology.* 2020;183(2):242-255.
3. Simpson EL, Sinclair R, Forman S, et al. Efficacy and safety of abrocitinib in adults and adolescents with moderate-to-severe atopic dermatitis (JADE MONO-1): a multicentre, double-blind, randomised, placebo-controlled, phase 3 trial. *Lancet.* 2020;396(10246):255-266.
4. Guttman-Yassky E, Blauvelt A, Eichenfield LF, et al. Efficacy and Safety of Lebrikizumab, a High-Affinity Interleukin 13 Inhibitor, in Adults With Moderate to Severe Atopic Dermatitis: A Phase 2b Randomized Clinical Trial. *JAMA dermatology.* 2020;156(4):411-420.
5. Paller AS, Siegfried EC, Thaci D, et al. Efficacy and safety of dupilumab with concomitant topical corticosteroids in children 6 to 11 years old with severe atopic dermatitis: A randomized, double-blinded, placebo-controlled phase 3 trial. *Journal of the American Academy of Dermatology.* 2020;83(5):1282-1293.
6. Reich K, Kabashima K, Peris K, et al. Efficacy and Safety of Baricitinib Combined With Topical Corticosteroids for Treatment of Moderate to Severe Atopic Dermatitis: A Randomized Clinical Trial. *JAMA dermatology.* 2020;156(12):1333-1343.
7. Silverberg JI, Toth D, Bieber T, et al. Tralokinumab plus topical corticosteroids for the treatment of moderate-to-severe atopic dermatitis: results from the double-blind, randomized, multicentre, placebo-controlled phase III ECZTRA 3 trial. *The British journal of dermatology.* 2021;184(3):450-463.
8. Wollenberg A, Blauvelt A, Guttman-Yassky E, et al. Tralokinumab for moderate-to-severe atopic dermatitis: results from two 52-week, randomized, double-blind, multicentre, placebo-controlled phase III trials (ECZTRA 1 and ECZTRA 2). *The British journal of dermatology.* 2021;184(3):437-449.
9. Kabashima K, Matsumura T, Komazaki H, Kawashima M, Nemolizumab JPSG. Trial of Nemolizumab and Topical Agents for Atopic Dermatitis with Pruritus. *The New England journal of medicine.* 2020;383(2):141-150.
10. Silverberg JI, Simpson EL, Thyssen JP, et al. Efficacy and Safety of Abrocitinib in Patients With Moderate-to-Severe Atopic Dermatitis: A Randomized Clinical Trial. *JAMA dermatology.* 2020;156(8):863-873.
11. Simpson EL, Forman S, Silverberg JI, et al. Baricitinib in patients with moderate-to-severe atopic dermatitis: Results from a randomized monotherapy Phase 3 trial in the United States and Canada (BREEZE-AD5). *Journal of the American Academy of Dermatology.* 2021.
12. Company ELa. A Long-term Study of Baricitinib (LY3009104) With Topical Corticosteroids in Adults With Moderate to Severe Atopic Dermatitis That Are Not Controlled With Cyclosporine or for Those Who Cannot Take Oral Cyclosporine Because it is Not Medically Advisable (BREEZE-AD4). https://clinicaltrials.gov/ct2/show/NCT03428100. Accessed March 1 2021.
13. Pfizer. Study Evaluating Efficacy and Safety of PF-04965842 and Dupilumab in Adult Subjects With Moderate to Severe Atopic Dermatitis on Background Topical Therapy (JADE Compare). https://clinicaltrials.gov/ct2/show/NCT03720470. Accessed March 1, 2021.
14. Sanofi. Evaluation of Dupilumab in Chinese Adult Patients With Moderate to Severe Atopic Dermatitis. https://clinicaltrials.gov/ct2/show/NCT03912259. Accessed June 23, 2021.
15. Pfizer. JAK1 Inhibitor With Medicated Topical Therapy in Adolescents With Atopic Dermatitis (JADE TEEN). https://clinicaltrials.gov/ct2/show/NCT03796676. Accessed June 25, 2021.
16. Guttman-Yassky E, Teixeira HD, Simpson EL, et al. Once-daily upadacitinib versus placebo in adolescents and adults with moderate-to-severe atopic dermatitis (Measure Up 1 and Measure Up

© 2022 Drucker AM, et al. *JAMA Dermatology*.

2): results from two replicate double-blind, randomised controlled phase 3 trials. *Lancet.* 2021;397(10290):2151-2168.
17. Reich K, Teixeira HD, de Bruin-Weller M, et al. Safety and efficacy of upadacitinib in combination with topical corticosteroids in adolescents and adults with moderate-to-severe atopic dermatitis (AD Up): results from a randomised, double-blind, placebo-controlled, phase 3 trial. *Lancet.* 2021;397(10290):2169-2181.
18. Merola JF, Bagel J, Almgren P, et al. Tralokinumab does not impact vaccine-induced immune responses: Results from a 30-week, randomized, placebo-controlled trial in adults with moderate-to-severe atopic dermatitis. *Journal of the American Academy of Dermatology.* 2021;85(1):71-78.

© 2022 Drucker AM, et al. *JAMA Dermatology*.
